# Anti-Amyloid Antibodies in the Treatment of Alzheimer’s Disease: An Umbrella Review

**DOI:** 10.3390/jcm15176782

**Published:** 2026-09-01

**Authors:** Stefania Kalampokini, Iraklis Keramidiotis, Antonis Frontistis, Francesca Zuchi, Dimitrios Michmizos, Vasileios Papaliagkas, Effrosyni Koutsouraki

**Affiliations:** 1 1st Department of Neurology, AHEPA University Hospital, 54636 Thessaloniki, Greece; antonisfront@gmail.com (A.F.); ekoutsou@auth.gr (E.K.); 2 Department of Neurosurgery, AHEPA University Hospital, 54636 Thessaloniki, Greece; dralkis@windowslive.com; 3 School of Medicine and Surgery, University of Rome Tor Vergata, 00133 Rome, Italy; francesca.zuchi@gmail.com; 4 Medical School of Aristotle, University of Thessaloniki, 54124 Thessaloniki, Greece; dimitrios.michmizos@gmail.com; 5 Department of Biomedical Sciences, School of Health Sciences, International Hellenic University, 57400 Thessaloniki, Greece; vpapal@auth.gr

**Keywords:** anti-amyloid antibodies (AAAs), Alzheimer’s disease (AD), efficacy, safety, ARIA, umbrella review

## Abstract

**Background:** Over the last decade, numerous studies have investigated the administration of monoclonal anti-amyloid antibodies (AAAs) as a therapeutic approach in Alzheimer’s disease (AD). The purpose of this umbrella review is to summarize current knowledge concerning the efficacy and safety of FDA- and EMA-approved AAAs for AD, namely, lecanemab and donanemab. **Methods:** We conducted a literature search in the PubMed, Scopus, and Web of Science databases in English, focusing on systematic reviews and/or meta-analyses, assessed by AMSTAR 2, concerning clinical and imaging efficacy, i.e., cognitive improvement and reduction of beta-amyloid on PET scan, as well as safety, i.e., adverse events and amyloid-related imaging abnormalities (ARIA). The extracted review-level dataset was entered into SPSS Version 26 for descriptive statistical analysis. **Results:** This umbrella review included 11 systematic reviews and/or meta-analyses. Patients treated with lecanemab or donanemab showed statistically significant changes on cognitive scales such as the Clinical Dementia Rating-Sum of Boxes (CDR-SB) (mean effect estimate −0.485, SD = 0.152, −0.70 to −0.34) and the Alzheimer’s Disease Assessment Scale-Cognitive Subscale (ADAS-Cog), but most were below or around clinically meaningful thresholds. Lecanemab and donanemab showed a statistically significant decrease in amyloid PET outcomes, including centiloids or the standardized uptake value ratio. Both antibodies were linked to ARIA, including amyloid-related imaging abnormalities-edema (ARIA-E OR 8.32–12.26) and amyloid-related imaging abnormalities-hemorrhage (ARIA-H OR 2–5.77), especially in ApoEε4 carriers. **Conclusions:** Lecanemab and donanemab are biologically active drugs for the treatment of early AD, with cognitive benefits below or around the clinically meaningful threshold. They are, however, associated with increased ARIA risk. Their administration depends upon careful patient selection, shared decision-making, and clear communication regarding expectations.

## 1. Introduction

Alzheimer’s disease (AD), which is one of the most prevalent neurodegenerative diseases leading to cognitive decline, affects an estimated 55 million people worldwide [1]. The burden of the disease is high; AD accounts for 60–70% of dementia cases, while it has been estimated that the number of patients will double by 2050 [2]. The disease begins from the asymptomatic stage with normal or abnormal AD biomarkers, i.e., amyloid deposition on amyloid PET or biomarkers in cerebrospinal fluid (CSF) such as Aβ42/40, p-tau181/Aβ42, and t-tau/Aβ42 (stage 0 or 1, respectively); and it then progresses to subjective mental disorders with normal cognitive testing, abnormal imaging biomarkers such as amyloid PET, and biomarkers in CSF or recently in plasma such as pTau217 [3,4,5], without impairment in daily functioning (stage 2). Stage 3 is characterized by abnormal cognitive tests and biomarkers but the maintenance of independent daily functioning; and then the disease progresses to mild (stage 4), moderate (stage 5) and ultimately severe dementia (stage 6) [6]. The current treatment for AD remains symptomatic and comprises cholinesterase inhibitors and NMDA receptor antagonists [7].

**Figure 1:**
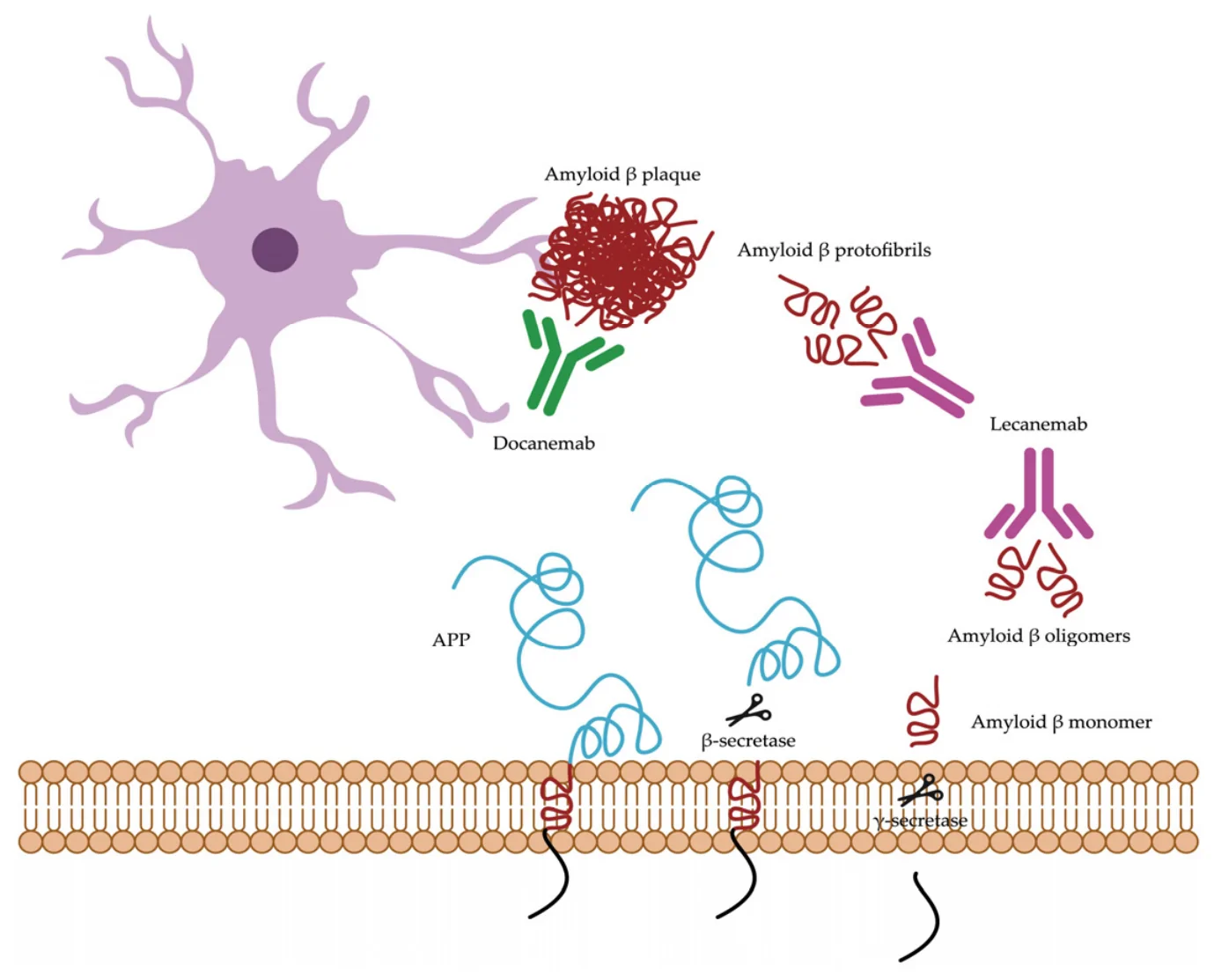
Mechanism of action of lecanemab and donanemab on beta-amyloid.

The main pathophysiological mechanism in AD lies in the inability to remove the extracellular accumulated beta-amyloid (Aβ), resulting in the formation of amyloid plaques in various brain areas [8,9]. Aβ contributes to cell death by interfering with neuron–neuron communication at synapses, while neurofibrillary tangles of Tau restrict the passage of essential nutrients inside neurons [9]. Anti-amyloid antibodies (AAAs) are promising agents that demonstrated marked reductions in amyloid plaque burden, as visualized by amyloid PET imaging, as well as statistically significant, albeit modest, slowing of cognitive decline in early-stage AD populations [10,11,12,13]. While first-generation AAAs target monomeric and fibrillary Aβ species, second- and third-generation AAAs are designed to recognize and neutralize aggregated Aβ species, such as protofibrils, fibrils, and dense-core amyloid plaques (Figure 1) [14,15,16]. AAAs can reduce parenchymal Aβ accumulation and Aβ-induced neuronal death in the brain through the following mechanisms: (a) by inhibiting the amyloid cascade—by specifically binding to Aβ aggregates, including fibrils and plaques, AAAs facilitate Aβ degradation to lower-order aggregates, prevent reassembly of Aβ, and thus inhibit Aβ-induced neurodegeneration; (b) by activating neuroinflammation, activating complement and inducing microglial-mediated phagocytosis of Aβ; and (c) by peripheral clearance of Aβ, the so-called “Peripheral Sink Hypothesis” [16,17].

So far, the FDA has approved three humanized IgG1 AAAs: aducanumab, lecanemab, and, most recently, donanemab [16]. Aducanumab received accelerated approval in June 2021 but was discontinued at the beginning of 2024 due to lack of evidence of meaningful clinical benefit and market challenges [18]. Lecanemab, sold under the brand name Leqembi^®^, was approved by the FDA in 2023 and by the EMA in April 2025. Lecanemab was evaluated in a large, 18-month phase II, double-blind study with an open-label extension phase [12], as well as in the phase III study (CLARITY AD) [10]. Lecanemab, which is administered intravenously every other week at a weight-adjusted dosage of 10 milligrams per kilogram of body weight [19], preferentially binds soluble Aβ protofibrils, with some activity on insoluble fibrils, which are a major component of brain amyloid plaques [14,20]. Donanemab, marketed as Kinsula^®^, was approved by the FDA in July 2024 and the EMA in July 2025 based on data from phase II and III clinical trials (mainly the TRAILBLAZER-ALZ 2 study [11]) after having received an initial rejection due to insufficient safety data [21]. Donanemab selectively binds to a truncated form of Aβ that is primarily found in plaques [14,20,21]. The recommended dosing for donanemab is 700 mg every four weeks for three doses, followed by 1400 mg every 4 weeks thereafter [22]. Both lecanemab and donanemab are labeled for the treatment of early symptomatic AD, including mild cognitive impairment (MCI) and mild dementia [21].

Monoclonal antibodies targeting Aβ have been linked to cranial MRI abnormalities, known as amyloid-related imaging abnormalities (ARIA), arising as a result of amyloid clearance [23]. While ARIA typically emerge early in treatment and are often asymptomatic, transient, or tend to resolve spontaneously over the course of several months [24], severe complications, including seizures and status epilepticus, can occur, though they are rare. There are two primary types of cranial MRI signal abnormalities associated with ARIA, which can present either independently or simultaneously: ARIA-edema/effusion (ARIA-E) and ARIA-hemosiderosis/hemorrhage (ARIA-H). ARIA-E arises from vasogenic edema or sulcal effusion within the leptomeningeal and subpial spaces and can be seen as hyperintense parenchymal or sulcal signals on MRI, predominantly involving cortical folds on T2-weighted and FLAIR sequences [24]. ARIA-H refers to microhemorrhages or larger hemorrhages, visible as hypointense hemosiderin deposits on imaging [21].

As the use of AAAs in AD is a topic of intense debate and clinicians will be challenged to communicate a new, rapidly evolving paradigm of AD care, this paper aims to evaluate the AAAs that have received FDA and EMA marketing approval, lecanemab and donanemab. The primary purpose of this paper was to evaluate the efficacy of AAAs, i.e., the change in cognitive function, as well as in amyloid PET imaging, using objective criteria, as well as their safety in relation to adverse effects, i.e., the frequency and severity of adverse effects, such as ARIA, to conclude whether their contribution to the treatment of AD is of clinical relevance. As the literature on this controversial issue is extensive, we conducted an umbrella literature search, which, to the best of our knowledge, is the first conducted so far, to summarize current knowledge from high-quality systematic reviews and meta-analyses referring to the effectiveness, adverse effects, and overall evaluation of AAAs in AD. Thus, this umbrella review aimed to address the uncertainty of the clinical relevance of the AAAs lecanemab and donanemab.

## 2. Materials and Methods

This review was conducted in accordance with the Preferred Reporting Items for Systematic Reviews and Meta-analyses (PRISMA) guidelines [25] (Supplementary Material Files S1 and S2) and guidance articles for umbrella reviews [26,27,28,29].

### 2.1. Protocol Registration

This review was registered in the PROSPERO online database of systematic review protocols (https://www.crd.york.ac.uk/prospero/, accessed on 7 January 2026) with protocol number CRD420251272807. This umbrella review can be categorized as an “intervention-specific umbrella review,” which explores the effects of specific interventions on health outcomes in a group of patients with a neurological disorder [29].

### 2.2. Eligibility Criteria

For this umbrella review, English-language peer-reviewed articles were assessed. We included systematic reviews and/or meta-analyses referring to the clinical and imaging efficacy and/or safety of lecanemab and donanemab in AD. Articles not written in English, animal or basic research studies, and other types of publications, such as conference reports and book chapters, were excluded.

### 2.3. Literature Search

We conducted a literature search in the PubMed (Medline), Scopus, and Web of Science databases from inception until May 2026 in English and in humans. The search strategy was as follows: (((((anti-amyloid antibodies) OR (lecanemab)) OR (donanemab) OR (anti-amyloid treatment) OR (amyloid-targeting)) AND (alzheimer’s disease)) AND (efficacy)) AND (((safety) OR (ARIA) OR (Amyloid-related imaging abnormalities)) OR (adverse events)).

### 2.4. Selection of Sources and Outcome Measures

**Figure 2:**
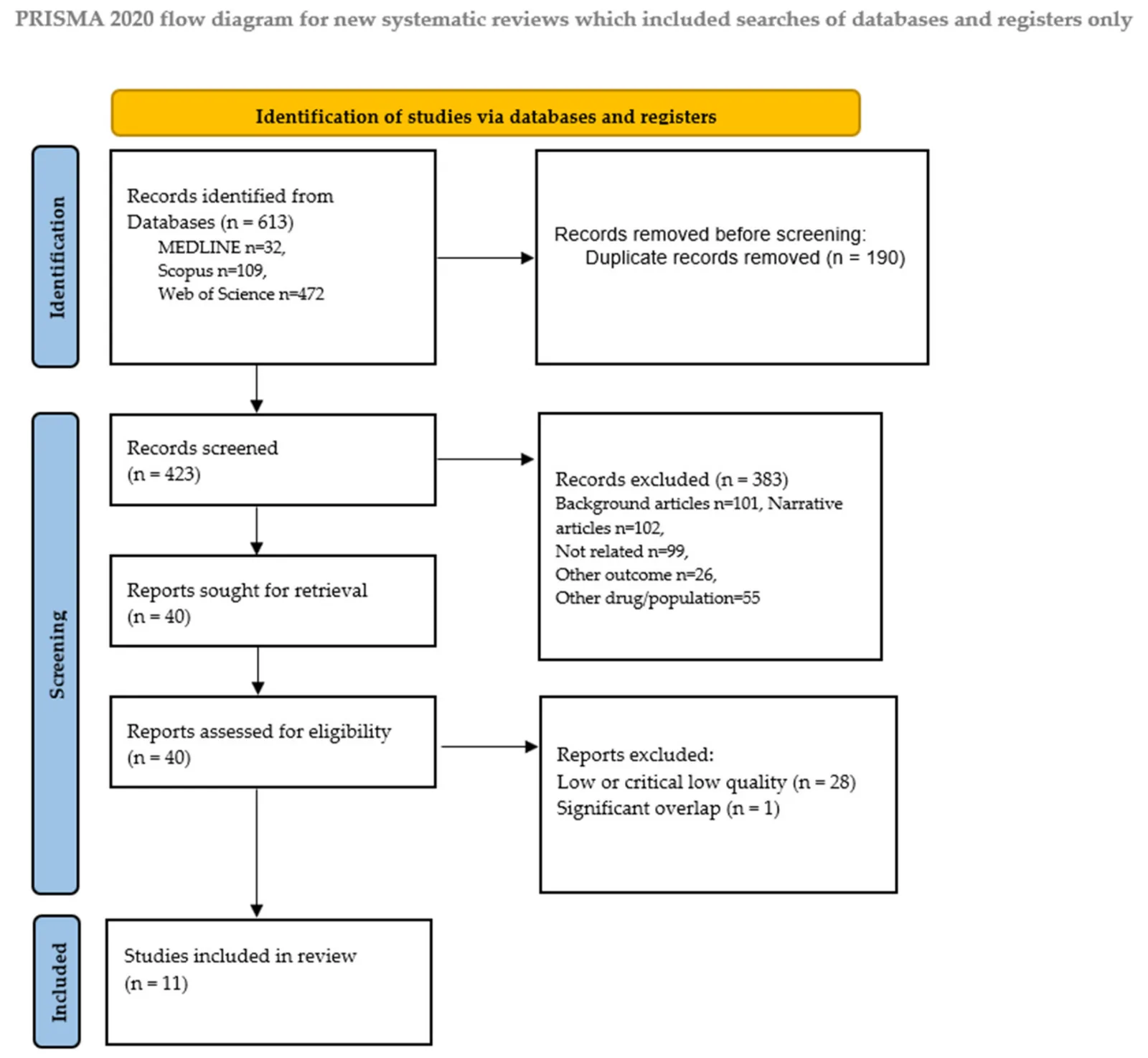
PRISMA flowchart of the included studies in the umbrella review.

The database search yielded 613 records, which were exported to the web tool Rayyan.ai. Following the identification and removal of duplicates, 423 articles remained for screening, in accordance with the eligibility criteria. After excluding articles unrelated to the topic, articles referring to other monoclonal antibodies, background articles, animal studies, non-systematic (narrative) reviews, and other publications (conference abstracts, book chapters, opinion articles), the remaining articles numbered 40. From those, only studies of moderate or high quality, as assessed using the AMSTAR 2 tool [30], were included. Thus, we ended up with 11 studies to include in the umbrella review [31,32,33,34,35,36,37,38,39,40,41]. Screening was conducted by two independent authors (SK, IK). Any differences were settled with the help of a third reviewer (AF). The complete selection process is shown in the PRISMA flow diagram in Figure 2. The characteristics of the included systematic reviews/meta-analyses can be seen in Supplementary Table S1.

The various primary studies included in the systematic reviews/meta-analyses used the following cognitive scales for assessing the patients’ cognitive change before and after the intervention with AAAs: MMSE (Mini-Mental Status Examination) [42]; ADAS-Cog (Alzheimer’s Disease Assessment Scale–Cognitive Subscale), most commonly ADAS-Cog13 and ADAS-Cog14, which are modifications of the initial ADAS-Cog11 scale [43]; CDR-SB (Clinical Dementia Rating Sum of Boxes) [44]; ADCOMS (Alzheimer’s Disease Composite Score) [45]; ADCS-ADL (Alzheimer’s Disease Cooperative Study—Activities of Daily Living) [46]; and Subjective Cognitive Decline (SCD) or Subjective Memory Decline (SMD), which refers to the self-perceived experience of worsening memory or cognitive function [47,48]. Imaging efficacy was evaluated based on amyloid PET parameters: centiloid change, standardized uptake value ratio (SUVR/SUVr) reduction, and amyloid clearance. Adverse events, serious adverse events, ARIA-E, ARIA-H, symptomatic ARIA, and discontinuation outcomes were used for synthesizing safety.

### 2.5. Data Extraction and Charting Process

To ensure consistency in data extraction, a data charting form was jointly developed by two authors (SK, EK). Extracted variables included publication information (name of the first author, year of publication, doi), type of study (systematic review with or without meta-analysis), studies included and clinical trials phase, studied AAA, dose of AAA, number of included patients, duration of treatment, main conclusions on clinical efficacy, imaging efficacy and safety of AAA (versus placebo), quality score (AMSTAR 2 scoring), heterogeneity and risk of bias. Data were extracted by four authors (SK, IK, AF, FZ) with discrepancies resolved through discussion until consensus was reached.

### 2.6. Quality and Risk of Bias Assessment

To document the methodological transparency and highlight the recurring limitations of the available literature, a critical appraisal of the included publications was conducted. The AMSTAR 2 is a critical appraisal tool for systematic reviews that include randomized or non-randomized studies of healthcare interventions, or both [30]. AMSTAR 2 evaluates the methodological quality of systematic reviews using 16 items/domains (7 critical, 9 non-critical) [30,49]. It focuses on creating a pattern of weaknesses/flaws in critical and non-critical domains to assign an overall rating as high, moderate, low, or critically low quality, rather than generating an overall score [49]. The AMSTAR 2 scores of the included and excluded articles after quality check can be seen in Supplementary Tables S2 and S3.

### 2.7. Assessment of Overlap of Studies

The systematic reviews/meta-analyses of our umbrella review included 22 primary studies assessing the safety and efficacy of lecanemab, donanemab, or both in clinical trial settings [10,11,50,51,52,53,54,55,56,57,58,59,60,61,62,63,64] or in real-world situations/centers [12,65,66,67,68]. The second stage of the process was de-duplication of the primary study evidence, since some clinical trials were prone to multiple counts in multiple systematic reviews/meta-analyses. The corrected covered area (CCA) approach was used as an objective method for quantification of the overlap of studies. A citation matrix was developed, with each review included and cross-tabulated to each different primary study. This enabled us to calculate the extent of overlap of the included meta-analyses and to create an overlap heatmap of the umbrella review. The extracted review-level data were used to map the outcome domains, effect directions, confidence intervals, reported heterogeneity, and overlap between primary studies. Because effect measures varied across outcomes and review-level estimates, descriptive statistics were calculated only within prespecified sets of estimates that used the same outcome scale. These statistics were used to describe the extracted review-level evidence and were not treated as newly pooled effects. A de novo meta-analysis of primary trials was not undertaken. The extracted review-level dataset was entered into IBM SPSS Statistics Version 26 for descriptive statistical analysis. The CCA calculations and visualization of overlap were performed in MS Excel because of the matrix-based structure of the overlap analysis. Separate analyses were conducted for clinical efficacy outcomes (CDR-SB/CDR-SOB and ADAS-Cog) that had sufficient data, imaging efficacy outcomes (Amyloid PET/SUVr), safety outcomes (ARIA-E and ARIA-H), and heterogeneity indices (I^2^). Descriptive statistics such as the number (N), minimum, maximum, range, mean, and standard deviation were computed for each outcome category, when applicable. Bar charts were used to display graphical representations of the distribution of reported effect estimates within the included reviews. Descriptive measures were used for different patient subgroups according to age, gender, ethnicity, and ApoE4 status, due to limited data. The synthesis did not solely count positive and negative reviews; rather, it assessed the consistency of findings, statistical significance, whether the results were clinically important, and whether the level of heterogeneity was low or considerable. This enabled the umbrella review to provide a balanced conclusion on the benefit–risk profile of lecanemab and donanemab.

## 3. Results

### 3.1. Methodological Quality and Overlap Analysis of Included Studies

**Figure 3:**
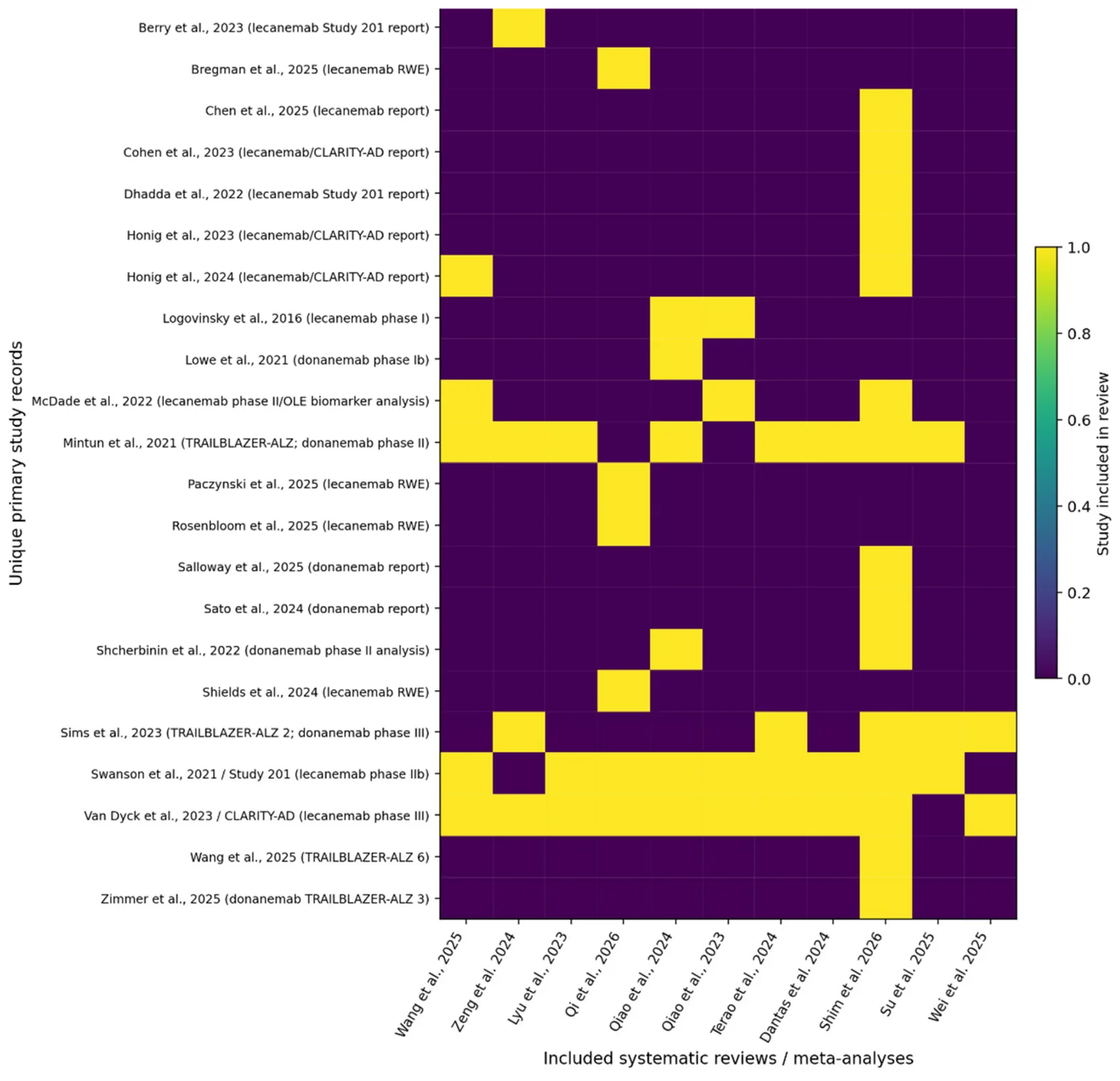
Overlap heatmap of studies included in the umbrella review. X axis: Berry et al. 2023 [55], Bregman et al. 2025 [65], Chen et al. 2025 [56], Cohen et al. 2023 [57], Dhadda et al. 2022 [63], Honig et al. 2023 [58], Honig et al. 2024 [59], Logovinsky et al. 2016 [60], Lowe et al. 2021 [50], McDade et al. 2022 [61], Mintun et al. 2021 [51], Paczynski et al. 2025 [66], Rosenbloom et al. 2025 [67], Salloway et al. 2025 [52], Sato et al. 2024 [53], Schcherbinn et al. 2022 [54], Shiels et al. 2024 [68], Sims et al. 2023 [11], Swanson et al. 2021 [12], Van Dyck et al. 2023 [10], Wang et al. 2025 [64], Zimmer et al. 2025 [62]. Y axis: Wang et al. 2025 [31], Zeng et al. 2024 [32], Lyu et al. 2023 [33], Qi et al. 2026 [34], Qiao et al. 2024 [35], Qiao et al. 2023 [36], Terao et al. 2024 [40], Dantas et al. 2024 [41], Shim et al. 2026 [37], Su et al. 2025 [38], Wei et al. 2025 [39]. RWE: real world evidence.

The methodological quality of the included reviews was moderate to high, using the AMSTAR 2 rating. Eight studies of high quality [34,35,36,37,38,39,40,41] and three of moderate quality [31,32,33] were included in the umbrella review. The overlap analysis resulted in 11 systematic reviews and meta-analyses and 22 unique primary-study records. The calculated CCA was 15%, meaning there was a high level of overlap of the included evidence. Figure 3 illustrates the degree of overlap between the included systematic reviews/meta-analyses and their underlying primary studies in a review–study overlap heatmap. Yellow cells indicate that the corresponding primary study was included in a review; dark purple cells indicate that the corresponding primary study was not included in a review. The rows that contain a large number of yellow cells indicate studies that have been included more than once in the reviews, and the columns that contain a large number of yellow cells indicate reviews that contain a large number of the eligible primary studies. The heatmap shows that several pivotal trials, especially the CLARITY-AD [10], Study 201 [12], and TRAILBLAZER-ALZ studies [11], were repeatedly included across multiple reviews.

### 3.2. Clinical Efficacy

**Figure 4:**
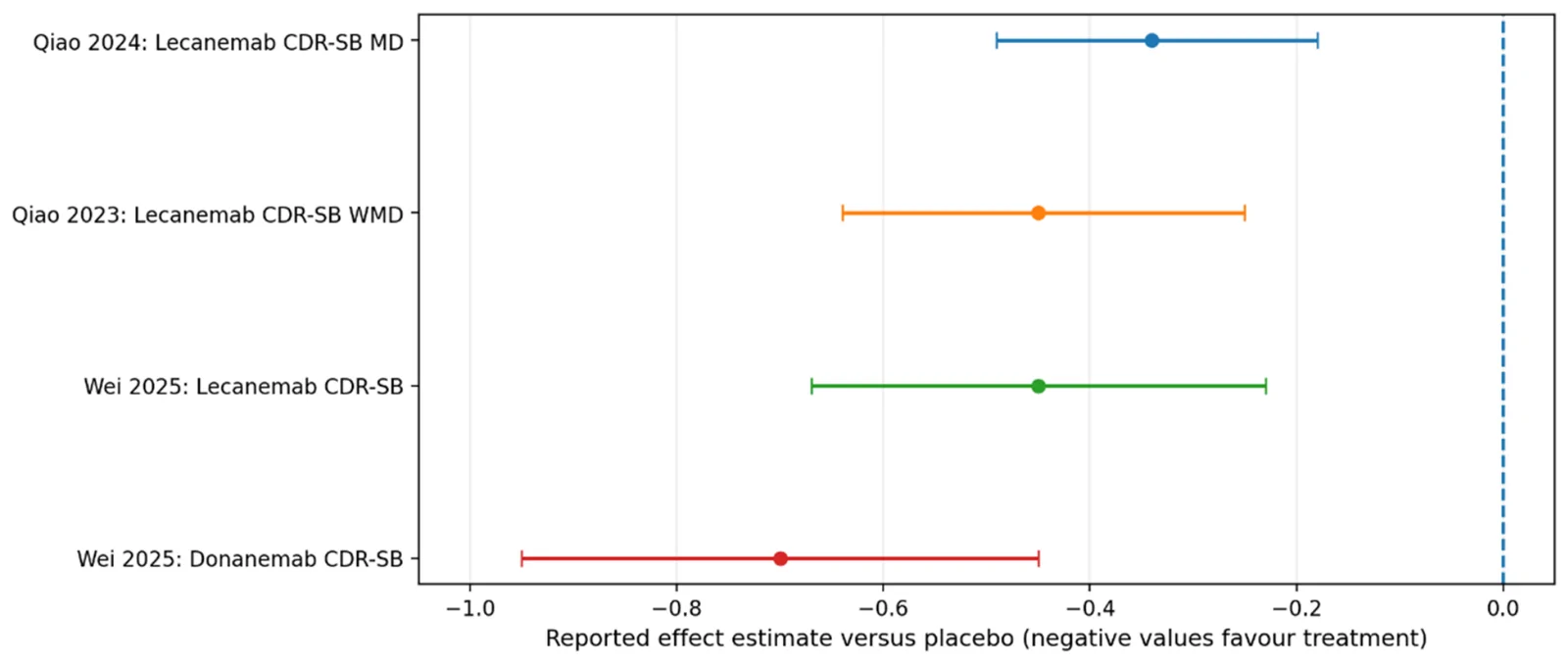
Clinical efficacy: reported review-level CDR-SB estimates for lecanemab and donanemab versus placebo [35,36,39].

The results of the included reviews suggest that both lecanemab and donanemab have a statistically significant clinical effect compared with placebo, especially within the early AD cohorts. Cognitive decline measured by the change in cognitive scales such as CDR-SB, ADAS-Cog, and ADCOMS was reduced consistently across the included reviews. The clinical efficacy of lecanemab and donanemab on the CDR-SB/CDR-SOB based on review-level estimates compared to placebo can be seen in Figure 4. Seven review-level CDR-SB estimates were identified after 18 months of treatment with lecanemab or donanemab. Treatment preference, indicated by negative values, is most common, and most estimates include confidence intervals without crossing the line of zero. The mean effect estimate was −0.485, (SD = 0.152), with values ranging from −0.7 to −0.34. The overall trend of negative estimates in all included reviews indicates that AAAs are likely to impact cognitive decline favorably.

For the ADAS-Cog, there were seven ADAS-Cog-related review-level estimates identified across ADAS-Cog, ADAS-Cog13, and ADAS-Cog14 outcomes. All seven reported estimates favored active treatment, and their confidence intervals remained on the treatment-favoring side of the null. However, their numerical magnitudes should not be directly compared due to the different outcome versions of the ADAS-Cog scale and effect metrics (Table 1). Table 1 presents these findings in a metric-separated evidence map. Thus, the majority of the CDR-SB and ADAS-Cog estimates preferred lecanemab or donanemab compared to placebo and were statistically significant, except for donanemab in two studies [33,38]. Overall, there is a uniform direction of clinical benefit, though the extent of the benefit/effect size is limited. Concerning the change in other cognitive scales, the results for the MMSE were reported as statistically significant in three included systematic reviews with mean reductions ranging from 0.48 to 1.4 points for patients treated with donanemab for up to 18 months [38,39], while no statistically important changes were reported for both lecanemab and donanemab in one review [40]. The mean reduction of the ADCOMS for lecanemab [36] was favorable at about −0.05 (95% CI: −0.07, −0.03). Lastly, the effect of lecanemab on the ADCS-ADL was favorable after up to 18 months [35], with a mean change of two, which is considered clinically relevant [69]. On the contrary, Su et al. reported a very small, though statistically significant, change for lecanemab-treated patients in the ADCS-ADL after 18 months. The effect on this scale was more pronounced in the primary study of lecanemab [10].

The clinical efficacy of AAAs in clinical trials, including patients with mild or early AD, was based on the changes in cognitive and functional scales, commonly the CDR-SB, ADAS-Cog, ADCOMS, MMSE, and, less frequently, the ADCS-ADL. In many included systematic reviews, the changes in the cognitive scales were, although statistically significant, not clinically meaningful for both lecanemab [33,35,36,37,38] and donanemab [38]. When using the CDR-SB, changes of 1.07–2.06 points are considered clinically meaningful for patients with MCI, 1.5–2.12 points in early AD, and 1.79–2.25 points in mild AD [70]. The reported treatment effects were, in contrast, generally less than 1 point for lecanemab and donanemab, suggesting that the change was statistically significant but not necessarily discernible at the patient level. A similar pattern was seen for the ADAS-Cog, with clinically meaningful change typically estimated around 2–5 or 3–4 points for the ADAS-Cog-13 and ADAS-Cog-14 scales, respectively [70,71,72]. The included reviews reported generally positive effects of treatments on the ADAS-Cog scale, though these effects were frequently below the above-mentioned clinically meaningful thresholds. Moreover, in the ADCOMS, proposed thresholds include a range of about 0.05 change in MCI and 0.1 in dementia [73]. In the review by Qiao et al., including primary studies mainly with patients with mild AD, the reduction in the ADCOMS in patients treated with lecanemab was only 0.05 [36]. On the other hand, the primary study of lecanemab reported a meaningful change of 0.164 in the treated group [10]. For the MMSE, the only clinically meaningful change was reported in one review paper for donanemab, with a mean change in the MMSE of 1.4 after 18 months [38]. Real-world data on efficacy outcomes of lecanemab or donanemab in AD in clinical practice are scarce. A recent paper showed that MMSE scores declined significantly over 6 months in 53 patients with early-stage AD, especially in younger patients, whereas no significant change was observed at 12 months for a smaller sample of those patients [65]. Overall, lecanemab and donanemab demonstrate statistically significant slowing of cognitive and functional decline on cognitive scales. However, in relation to thresholds for clinically meaningful change, the magnitude of their benefit seems to be small.

#### 3.2.1. Subgroups

##### AD Stage

The impact of the AAAs lecanemab and donanemab on CDR-SB and ADAS-Cog scores was more pronounced in patients at the preclinical/early stage of AD than in those at the mild-to-moderate stage [39].

##### Age

Regarding patient age, lecanemab showed significant slowing of cognitive decline in individuals aged 65 or older, while donanemab showed benefit across all ages, with the greatest benefit in patients aged between 65 and 74 years [37].

##### Gender

Lecanemab showed potential benefit primarily in male patients in the CLARITY-AD trial [37]. In contrast, donanemab demonstrated treatment benefits across both genders, suggesting that donanemab may be a more favorable treatment option for female patients [37].

##### Ethnicity

Lecanemab showed significant slowing of cognitive decline in Caucasian and Asian individuals, whereas donanemab had a similar effect in Caucasian, Asian, and African American individuals [37]. In the Clarity AD Asian region cohort, the overall efficacy and biomarker changes of lecanemab were consistent within the overall population, with a favorable risk–benefit profile [56].

##### ApoE4 Status

Donanemab reduced cognitive decline regardless of ApoE4 carrier status, i.e., was effective in slowing cognitive decline in both ApoEε4 carriers and non-carriers [37]. On the contrary, lecanemab showed no benefit in ApoEε4 homozygous carriers and a significant slowing of cognitive decline only in ApoEε4 heterozygous participants [37]. Therefore, it seems that ApoEε4 carriers, who have the highest genetic risk for the disease, derived reduced clinical benefit compared to non-carriers.

### 3.3. Imaging Efficacy

Concerning imaging efficacy, lecanemab and donanemab have been shown to significantly reduce amyloid burden compared with placebo across all reviews that addressed this matter [35,36,74]. In the different measures used to assess amyloid burden change on amyloid PET, i.e., centiloid reduction, standardized uptake value ratio (SUVR/SUVr) changes, or amyloid clearance, treatment with lecanemab [36] or donanemab [35] was associated with significant changes. The mean reduction between the donanemab and placebo groups was approximately −0.37 (SD 20.31, 95% CI −0.57, −0.17) in amyloid PET SUVr [35]. Across studies, it was clear that AAAs induced a very large reduction in amyloid burden on PET scans, which was moderately correlated with clinical improvements [35,36]. In a comparative meta-analysis among AAAs (aducanumab, lecanemab, donanemab, solanezumab, gantenerumab), donanemab showed the greatest benefit on amyloid PET SUVr [35]. However, there was considerably greater heterogeneity for imaging outcomes than for clinical scales [31,36].

Three imaging estimates at the review level were identified and are presented separately in Table 2. Two were the SUVr, and the one estimate was amyloid burden on PET. All point estimates were in favor of active treatment, but the 95% CI for lecanemab SUVr values was close to the null value. There were no pooled estimates for the mean, standard deviation, range, minimum, or maximum of amyloid burden measured by PET and calculated SUVr, as these are different PET-derived outcome measures, and they are expressed on different numerical scales.

The prime imaging evidence for lecanemab was from CLARITY-AD and Study 201 [10]. On PET, the amyloid burden showed a mean reduction of about 55.48 centiloids in the lecanemab group and a mean increase of 3.64 centiloids in the placebo group in CLARITY-AD [10]. Moreover, a considerable percentage, i.e., 65% and 81% of patients who received lecanemab, were reported to be amyloid-negative at 12 months and 18 months, respectively [61]. Concerning donanemab, the florbetapir PET results of the donanemab versus placebo groups in the primary study by Mintun et al. exhibited a strong treatment effect, with a greater reduction in amyloid plaques in the donanemab group [51]. The percentage of participants in the donanemab group with amyloid-negative status at 24, 52, and 76 weeks was 40%, 59.8%, and 67.8%, respectively [51]. In the TRAILBLAZER-ALZ randomized controlled trial (RCT) study, the rate of donanemab-induced amyloid reduction at 6 months was moderately correlated with the amount of baseline amyloid [11]. However, participants with greater baseline amyloid levels, despite experiencing greater plaque reduction, had a lower probability of reaching the complete amyloid clearance threshold [54]. Donanemab slowed tau accumulation in a region-dependent manner as measured using neocortical and regional standardized uptake value ratios. More specifically, donanemab slowed tau accumulation in the temporal, parietal, and frontal lobes over 76 weeks [54].

### 3.4. Comparative Efficacy of Lecanemab and Donanemab

Both lecanemab and donanemab have a similar overall profile of statistically significant slowing of cognitive decline rate. The distinctions among them are largely quantitative and not qualitative. Lecanemab has a strong evidence foundation, primarily from CLARITY-AD and Study 201, and exhibited significant clinical impacts in the CDR-SB, ADAS-Cog/ADAS-Cog14, ADCOMS, and ADCS-ADL outcomes across included systematic reviews [33,35,36,39]. However, the extent of benefit was relatively small and not always clinically relevant. There was also a clear imaging efficacy seen in patients treated with lecanemab [36]. Donanemab also exhibited statistically significant clinical efficacy, including the CDR-SB and ADAS-Cog/ADAS-Cog13 measures [33,39], and exhibited robust imaging efficacy with significant clearance of amyloid [35]. In some network comparisons, donanemab was included in the highest proportion of clinically effective AAAs, particularly in terms of cognitive-functional slowing and amyloid reduction [38]. Therefore, this umbrella review does not lead to a straightforward conclusion of superiority of one antibody over the other.

In a network meta-analysis including 59 randomized controlled trials with a total of 18 interventions, including various AAAs, symptomatic treatments, plasma exchange, or placebo, assessing comparative efficacy, immunotherapeutic agents demonstrated a statistically better performance compared to symptomatic drugs for the primary efficacy outcome (CDR-SB scores) [38]. More specifically, donanemab and lecanemab ranked in the top positions based on changes in the CDR-SB, ADCS-ADL, MMSE, and ADAS-Cog scales [38]. In the MCI to mild dementia subgroups, donanemab and lecanemab significantly reduced CDR-SB decline compared to symptomatic drugs [38]. However, the authors report that only donanemab outperformed symptomatic drugs. Concerning the comparison between AAAs on cognitive scales (CDR-SB, ADCS-ADL, ADAS-Cog), donanemab had a slightly more favorable profile compared to lecanemab [38]. In another comparative meta-analysis, donanemab was associated with the greatest benefit on the ADAS-Cog, while lecanemab exhibited the highest efficiency on the ADCS-ADL [35]. In another network meta-analysis, short-term aerobic exercise and melatonin were found to be significantly more effective and tolerable than donanemab or lecanemab and had a faster onset of effect in patients with MCI and mild AD [40]. Those reviews that specifically studied AAAs compared to placebo did tend to show statistically significant differences; however, a larger comparison or a ranking of different interventions was less optimistic regarding the size and significance of the intervention [40].

### 3.5. Safety and ARIA Outcomes

The most frequently reported adverse events of lecanemab and donanemab were ARIA-E, ARIA-H, and infusion-related reactions.

#### 3.5.1. Amyloid-Related Imaging Abnormalities (ARIA)

**Figure 5:**
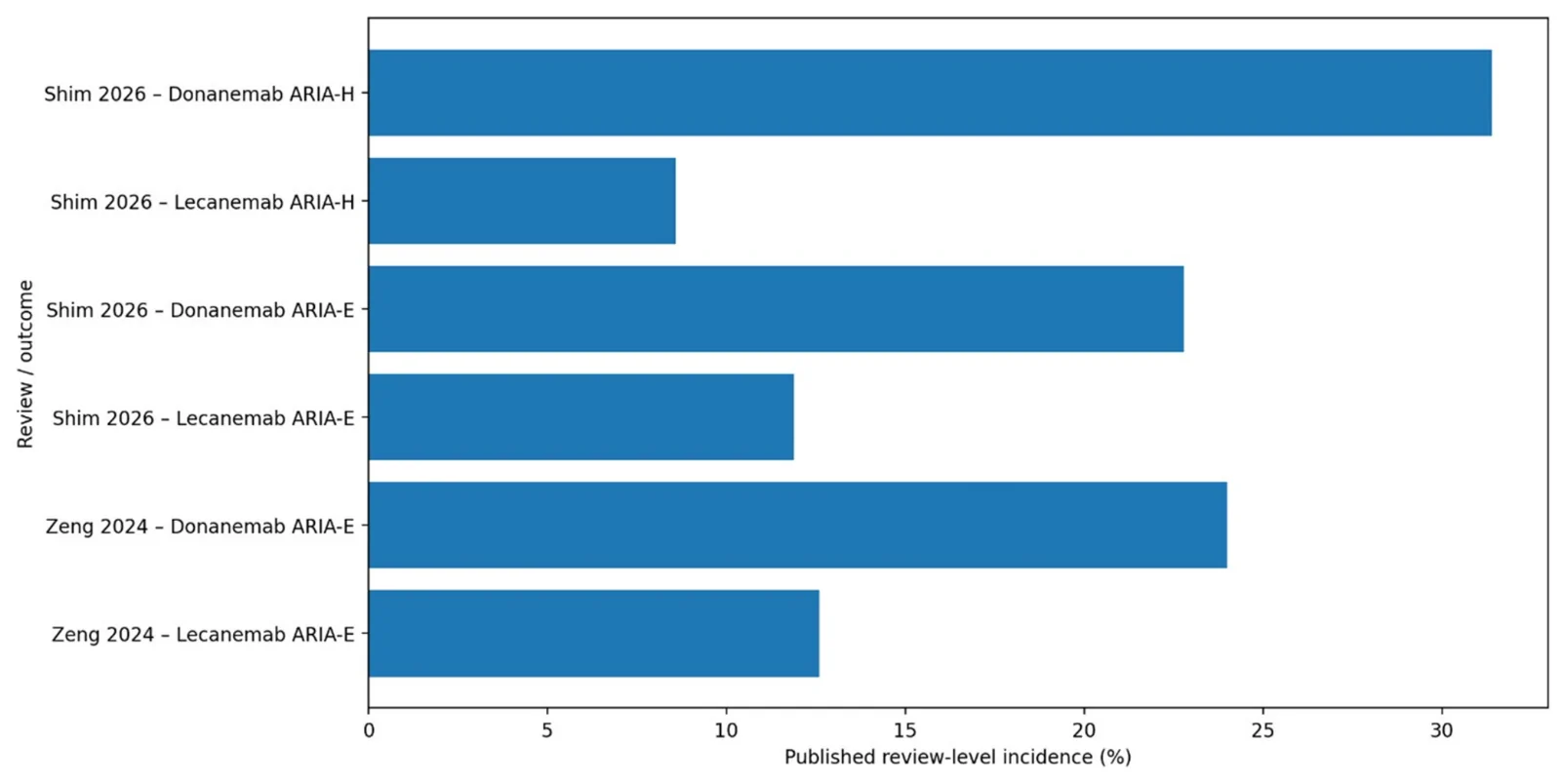
Published review-level ARIA incidence estimates [32,37].

Treatment with AAAs significantly increases the risk of both ARIA-E and ARIA-H. There were six risk estimates reported for ARIA-E and ARIA-H. All estimates were above the null value of 1.0, suggesting a higher risk of ARIA-E with active treatment than with placebo. The estimates for ARIA-E (OR) ranged from 8.32 to 12.26. This is a moderate to large relative safety effect, with a risk of ARIA-E of 8 to approximately 12 times higher than placebo. Concerning the ARIA-H risk, all estimates were found to be greater than 1.0, suggesting that there was an increase in the ARIA-H risk with active treatment relative to placebo. The estimates for ARIA-H (OR) ranged from 2 to 5.77. Therefore, the relative increase in risk was larger for ARIA-E than for ARIA-H. The estimates for ARIA-E and ARIA-H were based on partially overlapping reviews of the same pivotal trials. Published review-level ARIA-E and ARIA-H relative-effect estimates for lecanemab and donanemab versus placebo can be seen in Table 3 and Figure 5.

In the original studies, such as the phase III CLARITY-AD trial, ARIA-E and ARIA-H occurred in 12.6% and 17.3% of participants receiving lecanemab, respectively [10]. In the phase III TRAILBLAZER-ALZ-2 trial, ARIA-E and ARIA-H occurred in 24% and 31% of participants receiving donanemab, respectively [11]. Across these trials, ARIA were most often asymptomatic or mildly symptomatic and detected through routine MRI monitoring [75]. However, symptomatic ARIA, which can require hospitalization, specialized management, and prolonged monitoring, have been reported [11,19,22,76,77,78] and, in rare cases, have resulted in death [11,19,22,76,78,79]. Notably, symptomatic ARIA-E and ARIA-H were less frequent than total ARIA outcomes but remained clinically important. The available review-level estimates were based on few events and overlapping pivotal trials for symptomatic ARIA. For lecanemab, symptomatic ARIA-E was reported with an RR of 51.95 (95% CI: 3.17–851.95), while symptomatic ARIA-H was reported with an RR of 3.06 (95% CI: 0.62–15.10) [31]. The values when the open-label data for lecanemab were added were an RR of 50.94 (95% CI: 3.11–835.50) and an RR of 5.49 (95% CI: 1.22–24.72) for symptomatic ARIA-E and ARIA-H, respectively [31]. For donanemab, symptomatic ARIA-E was reported with an RR of 7.69 (95% CI: 0.98–60.64) and symptomatic ARIA-H with an RR of 2.65 (95% CI: 0.86–8.09) [31].

There was significant variation in the safety outcomes. Specifically, Qi et al. reported I^2^ = 42.3% for overall ARIA, I^2^ = 54.4% for ARIA-E, and I^2^ = 86.2% for ARIA-H [34]. Dantas et al. also reported high heterogeneity for ARIA-H (I^2^ = 87%) [41]. This suggests that the estimates of safety depend upon the characteristics of the study population, the definitions of ARIA, ApoE4 carrier status, the presence of cerebral microhemorrhage at baseline, the dose of the drug, and whether it has been used in a real-world setting or a trial. Indeed, real-world evidence from 234 patients in the USA treated with lecanemab reports that 22% of patients suffered ARIA, 15% ARIA-E, and 6.7% ARIA-H over 14 months [66]. Moreover, other real-world data reported that in a cohort of 71 patients treated with lecanemab, 24% suffered ARIA, most of whom were asymptomatic [68].

##### Risk Factors for ARIA

The most important risk factors for ARIA include exposure to AAAs, ApoE-e4 allele carrier status, higher drug doses, older age, elevated arterial pressure, and pre-existing cerebral microhemorrhages [80,81]. The risk of ARIA exhibits a clear gene-dose-dependent association with the ApoEε4 allele [34]. Specifically, carriers of the ApoEε4 allele were at an increased risk of ARIA. The risks of ARIA-E and ARIA-H were 2.2 times and 3.5 times higher in ApoEε4 carriers than in non-carriers, respectively [37]. Among carriers, homozygous carriers had a markedly higher prevalence of ARIA (ARIA-E: 40%; ARIA-H: 46%) compared to heterozygous carriers (ARIA-E: 17%, ARIA-H: 22%) [37].

#### 3.5.2. Adverse Events and Infusion-Related Reactions

Lecanemab and donanemab appeared to be associated with more adverse events than placebo [35,38,40,41]. In the CORE lecanemab study and its open-label extension, 15% of patients experienced serious adverse events [10]. Infusion reaction symptoms after the administration of AAAs typically included fever, chills, headache, rash, nausea, vomiting, abdominal discomfort, and elevated blood pressure [10]. The majority of infusion-related reactions (approximately 75%) occurred during the first infusion [10,12]. Real-world data from 71 patients treated with lecanemab reported that 37% experienced infusion reactions after their first lecanemab infusion, with headaches and chills being the most common adverse events [68]. The vast majority of patients (88%) reported side effects either at the infusion center or within the first 24 h post-infusion [68]. The substantial heterogeneity across studies appeared to be largely attributable to variations in management strategies, particularly the administration of prophylactic medication before the infusion [34].

#### 3.5.3. Deaths/Fatalities

There were nine deaths during the open-label extension of lecanemab, with four deemed possibly related to study treatment [10]. Three were attributed to intracranial hemorrhage, combined with anticoagulant therapy or tissue plasminogen activator [10,59]. The occurrence of deaths was similar regardless of ApoEε4 genotype [59]. Another three deaths were reported as drug-related, one due to multiple cerebral hemorrhages in combination with the administration of rt-PA [76], one due to inflammatory cerebral amyloid angiopathy in a homozygous ApoEε4 carrier [78], and one due to a myocardial infarction shortly after the first lecanemab infusion, although the latter was not certainly related to lecanemab treatment [68]. Twenty-two deaths were registered in the FDA Adverse Event Reporting System for lecanemab, mainly attributed to cardiac disorders, nervous system disorders, or neoplasms [82,83]. Donanemab was associated with three deaths due to ARIA, two in ApoE34 heterozygous carriers and one in a non-carrier [11].

#### 3.5.4. Comparative Safety of Lecanemab and Donanemab

Safety-wise, a few reviews indicated that lecanemab might have a lower ARIA burden than donanemab. This was most clearly seen in Zeng et al., reporting a lower incidence of ARIA-E for lecanemab versus donanemab (12.6% vs. 24%) [32]. Moreover, Shim et al. reported a lower pooled incidence of ARIA for lecanemab than for donanemab (11.9% vs. 22.8% for ARIA-E and 14.2% vs. 28.7% for ARIA-H, respectively) [37]. The same study reported that infusion-related reactions were markedly elevated with lecanemab [37]. Qiao et al. also reported a higher incidence for both ARIA-E and ARIA-H in patients treated with donanemab than in those treated with lecanemab (ARIA-E OR 12.26 vs. 8.32, ARIA-H 5.77 vs. 2.12) [35]. Notably, the comparisons are not direct, as each treatment group was compared to placebo. However, in the same comparative meta-analysis, lecanemab showed the lowest safety, in terms of overall adverse events [35]. Donanemab was also found to have a slightly more favorable profile compared to lecanemab (based on the P score) in another comparative network meta-analysis [38]. Death and serious adverse events did not seem to differ significantly between the two AAAs [32,37]. Concerning different subgroups of patients, ARIA were more prevalent in all subgroups of donanemab than lecanemab users, except for ARIA-H in ApoEε4 non-carriers, where no difference was observed between the two drugs [37]. Moreover, ARIA events and infusion-related reactions occurred less commonly with lecanemab in patients from Asian regions (Japan, Singapore, and South Korea) [37,56]. Lastly, the incidence of adverse events was higher in patients in the early/preclinical stage of disease compared to those in the mild to moderate/prodromal stage for both AAAs [39]. Notably, donanemab may appear to have a higher ARIA burden (both ARIA-E and ARIA-H) than lecanemab, but direct comparisons should be interpreted with caution due to differences in study populations, amyloid/tau selection criteria, stopping rules, and differences in MRI surveillance protocols.

### 3.6. Risk of Bias and Heterogeneity

Concerning the risk of bias, the core randomized trials of lecanemab and donanemab were rated as low or low-to-moderate risk of bias studies, most commonly using the Cochrane RoB 2 tool [84], while real-world evidence was assessed using tools such as the Joanna Briggs Institute (JBI) Critical Appraisal Checklist [85]. However, some concerns were reported for specific primary studies [10,12,51,52] in the included systematic reviews [37,38,39,40].

**Figure 6:**
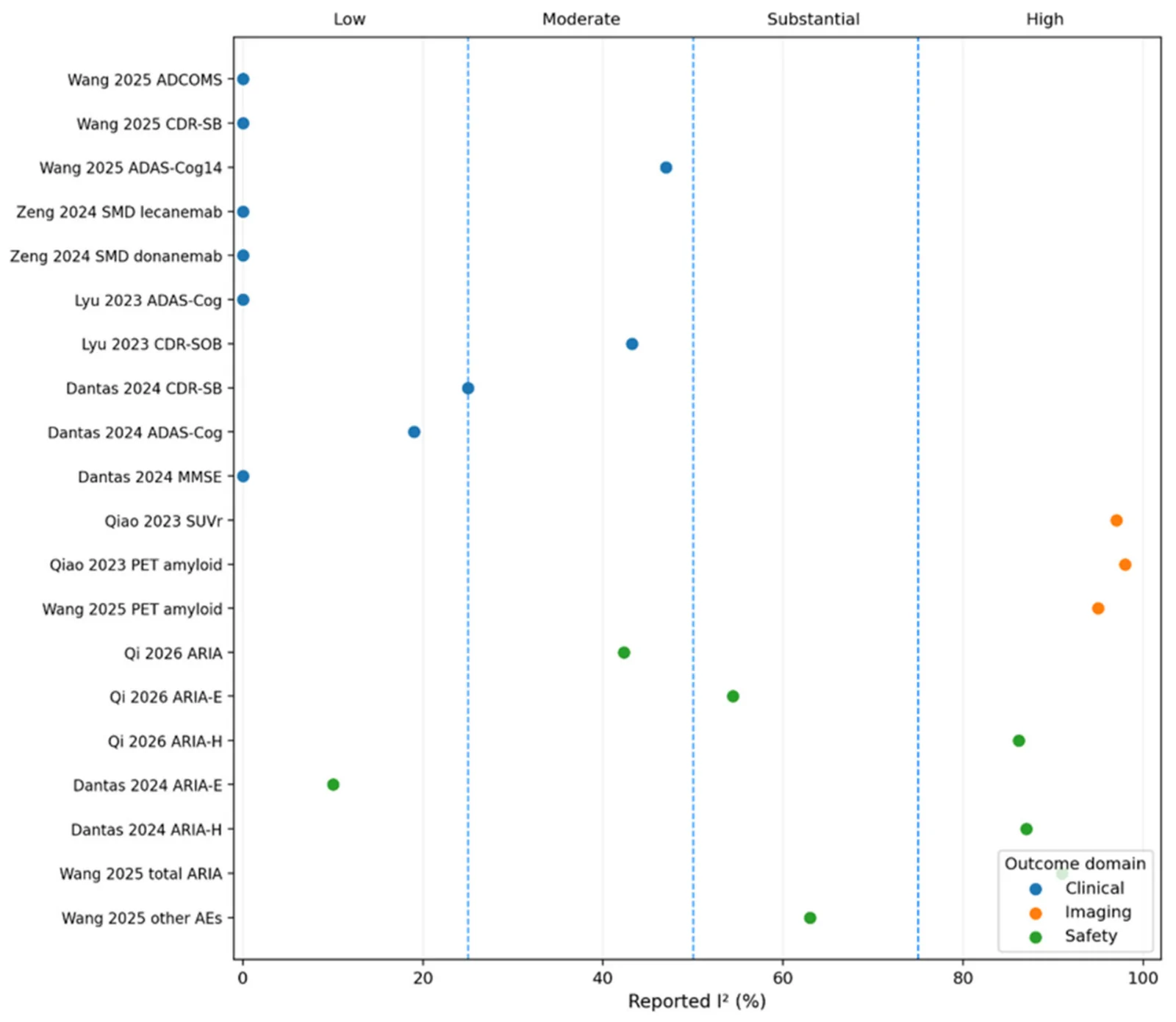
Descriptive review-level scatter plot of reported heterogeneity and statistical significance [31,32,33,34,36,41]. Abbreviations: ARIA-H: amyloid-related imaging abnormalities-hemorrhage/hemosiderosis, ARIA-E: amyloid-related imaging abnormalities-edema, PET: positron emission tomography, SUVr: standardized uptake value ratio on amyloid PET, AEs: adverse events, MMSE: Mini-Mental Status Examination, ADAS-Cog: Alzheimer’s Disease Assessment Scale–Cognitive Subscale, CDR-SB: Clinical Dementia Rating Sum of Boxes, SMD: subjective memory decline, ADCOMS: Alzheimer’s Disease Composite Score. Dashed lines indicate the boundaries for low, moderate, substantial, and high heterogeneity, respectively.

There was significant between-outcome domain variation in heterogeneity. There were 20 estimates of I^2^ obtained from the reviews included. Across the 20 extracted outcome-specific I^2^ values, the mean heterogeneity was 42.91% (SD 38.65%), with a reported range between 0% and 98%, which highlighted the substantial heterogeneity across the reviews. Most clinical outcomes had low to moderate heterogeneity, while the imaging and safety outcomes were more heterogeneous. Specifically, there was no heterogeneity in some cognitive scales, such as the MMSE in Dantas et al. [41], the ADCOMS and CDR-SB in Wang et al. [31], as well as the SMD in Zeng et al. [32]. Conversely, some ARIA outcomes were very heterogeneous, such as ARIA-H (86.2%) in Qi et al. [34], ARIA-H (87%) in Dantas et al. [41] and total ARIA (91%) in Wang et al. [31]. Similarly, the outcomes for amyloid PET were heterogeneous, including 97% for SUVr and 95–98% for amyloid burden [31,36]. The scatter plot in Figure 6 provides a visual comparison of the reported heterogeneity. Values of I^2^ close to 0% indicate low heterogeneity, while values between 50% and 75% and over 75% indicate moderate and high heterogeneity, respectively. The colors indicate the outcome domains: clinical, imaging, and safety. The vertical reference lines are set for moderate and high levels of heterogeneity thresholds. The figure reveals that the clinical and safety outcomes were statistically significant with low heterogeneity, while the imaging and adverse event outcomes had high heterogeneity. This supports the decision to interpret the findings narratively rather than as a simple pooled umbrella estimate. It also reinforces the need to consider differences in population characteristics, drug dose, trial design, imaging method, and follow-up duration.

## 4. Discussion

This umbrella review of previously published systematic reviews and meta-analyses on efficacy and safety measures of lecanemab and donanemab shows overall consistency with the direction of effect of these two approved AAAs. Lecanemab and donanemab are biologically and clinically active AAAs in MCI and early AD, although their overall benefit must be weighed against the risk of adverse events and especially ARIA. In clinical and imaging outcomes, there is a statistically significant benefit for both antibodies over placebo. Specifically, the evidence shows that there is a statistically significant deceleration of cognitive decline, which is particularly noticeable in the CDR-SB, ADAS-Cog and ADCOMS outcomes. Clinical outcomes tended to have low to moderate levels of heterogeneity, indicating that there was a relatively consistent direction of clinical benefit in many analyses. However, the clinical benefit seems to be limited and below or around clinically meaningful thresholds [70,71,72,86]. Assuming a linear progression, a patient taking lecanemab would need 3 to 6 years of treatment to reach the minimum threshold of a noticeable clinical difference [87]. The evidence is stronger on the efficacy of AAAs on brain imaging, as lecanemab and donanemab decrease amyloid burden compared to placebo. This is evidenced by centiloid reduction, SUVr change, and amyloid clearance on brain MRI [35,36]. Moreover, the evidence is clear concerning safety, as there is an increased risk of ARIA in patients treated with lecanemab or donanemab. In the reviews included, the ARIA-E and ARIA-H prevalence were found to be elevated compared to placebo, and the magnitude of the ARIA rates seems to be generally higher for donanemab than for lecanemab [32,35,37,88].

In terms of clinical efficacy in various patient groups, concerning the various patient subgroups, although the data are limited, it seems that donanemab is effective in every age group, with greater efficacy in patients between 65 and 74 years, while lecanemab is more effective in patients over 65 years [37]. Moreover, donanemab is reported to have the same efficacy in both genders, while lecanemab seems to be more effective in male patients [37]. As far as ethnicity is concerned, lecanemab seems to yield significant slowing of cognitive decline mainly in Caucasians and Asians, while donanemab has a beneficial influence on slowing cognitive decline in all ethnicities, including African Americans [37]. Lastly, donanemab reduced cognitive decline regardless of ApoE4 carrier status, i.e., was effective in slowing cognitive decline in both ApoEε4 carriers and non-carriers, whereas lecanemab showed no benefit in ApoEε4 homozygous carriers and a significant slowing of cognitive decline only in ApoEε4 heterozygous participants [37].

Original RCTs concentrated more on the effect of AAAs on cognition scales rather than quality of life or caregiver burden. Lecanemab was associated with relative preservation of Health-Related Quality of Life (HRQoL) and a decrease in caregiver burden, with consistent benefits across different quality of life scales and within-scale subdomains [57]. Statistically significant improvements in HRQoL favoring lecanemab were observed in participants aged 65–74 years and those with ApoEε4 heterozygous status [37]. No significant differences were found for African American, Asian, and Hispanic participants, nor in other subgroups for the EuroQol Visual Analogue Scale (EQ-VAS) or Quality of Life in Alzheimer’s Disease (QOL-AD) [37]. Indeed, secondary measures of efficacy such as time-to-event analysis and caregiver burden are important factors to be considered when assessing the clinical significance of AAAs. The real effect of positive results on the quality of life of AD patients would be a delay of progression, i.e., 6 months of preservation, from MCI to mild AD dementia or a delay from mild to moderate AD dementia. Notably, the main interest of the patients and their relatives is the effect of treatment on quality of life and not on laboratory data or biomarkers [89]. In this context, it may be necessary to implement appropriate questionnaires or scales that assess quality of life rather than solely assessing memory impairment when collecting real-world data.

Another issue is the benefit of the new anti-amyloid therapies compared to traditional agents on the management of dementia and AD. Short-term aerobic exercise and melatonin were found to be more effective, tolerable, and acceptable than donanemab and lecanemab on cognitive function in people with MCI and mild AD [40]. The superiority of exercise and melatonin over AAAs may be because the latter only act on Aβ, whereas exercise and melatonin have broader targets, such as inhibition of the abnormal phosphorylation of tau protein, anti-inflammatory effects, and the production of brain-derived neurotrophic factor [40]. Moreover, in terms of pharmaco-economic outcomes, the traditional treatments have a financial benefit for national health systems. Indeed, there is an ongoing global debate regarding the socioeconomic value of AAAs, especially in low- or medium-income countries. These drugs cost between $26,500 and $28,200 annually and require regular MRI monitoring, creating an enormous financial burden that patients or caregivers cannot actually perceive [90,91].

The imaging findings of the effect of AAAs were more robust than the clinical findings. The amyloid load on PET and SUVr-related outcomes revealed a decrease in amyloid burden for both AAAs [31,35]. Both lecanemab and donanemab demonstrated significant amyloid reduction, with a shift to an amyloid-negative state in a considerable percentage of patients, i.e., approximately 65% and 80% after 12 and 18 months, respectively, for lecanemab [61] and 60% and 70% over similar periods for donanemab [51]. The reduction in brain Aβ levels is indicative of the pharmacodynamic activity of both antibodies and supportive of effective amyloid target engagement. Although amyloid clearance is a good biomarker of drug action, whether it was proportional to a clinical benefit in cognition or function was not investigated. Only one primary study showed a strong correlation between the plasma Aβ42/40 ratio and ADAS-Cog14 for lecanemab [61]. Furthermore, another recent meta-analysis highlighted that brain volume loss (ventricular enlargement and hippocampal atrophy) after approximately 20 months was substantially greater in patients treated with AAAs (lecanemab, donanemab or aducanemab) than that typically observed with age-related atrophy in untreated patients [92].

In terms of safety, ARIA-E and ARIA-H were consistently increased in treated individuals with both lecanemab and donanemab compared to placebo groups. In our analysis, the relative increase in risk was greater for ARIA-E (approximately 8–12 times higher than placebo) compared to ARIA-H (2–6 times higher than placebo). The severity of ARIA-E and ARIA-H is determined by the size and distribution of FLAIR hyperintensities or cerebral microbleeds and superficial siderosis on MRI, respectively [19]. Specifically, a single hyperintense lesion under 5 cm, fewer than four microhemorrhages, or a single region of superficial siderosis are considered mild ARIA; hyperintensities measuring between 5 and 10 cm or involving multiple discrete regions, 5–9 microhemorrhages, or two areas of superficial siderosis are stratified as moderate; while any lesion exceeding 10 cm, ten or more microhemorrhages or more than two foci of siderosis are categorized as severe [19]. Lastly, the risk of adverse events (apart from ARIA) under treatment with AAAs was reported to be twice as high as placebo in one network meta-analysis [35], while other authors reported no difference in adverse events [32,36,38]. However, it was three times more likely for patients to discontinue treatment due to adverse events than for patients receiving placebo [74]. The majority of infusion-related reactions after the administration of AAAs were mild or moderate and typically included fever, chills, headache, rash, nausea, vomiting, abdominal discomfort, and elevated blood pressure [21,93]. Moreover, the vast majority occurred during the first infusion [10,12], underscoring the importance of extended post-infusion monitoring for 2–3 h during the initial dosing sessions. Although there was no reported difference in mortality between AAA treatment and placebo [31,32], at least seven and three deaths, to the best of our knowledge, have been attributed to treatment with lecanemab and donanemab, respectively, so far [10,11,68,76,78]. The majority of the few real-world data that are available on safety come from the FDA Adverse Event Reporting System database, i.e., twenty-two reported deaths mainly attributed to cardiac disorders, nervous system disorders, or neoplasms [94], rely on self-reporting and lack independent verification. Therefore, serious outcomes such as death may be misclassified or underreported, resulting in an incomplete classification of the severity and nature of these events [83]. Notably, imaging outcomes, and mainly ARIA, had considerable heterogeneity in the included meta-analyses. Some authors [36,39] reported heterogeneity values close to 0% for certain ARIA outcomes, while others [34,41] reported significant heterogeneity (particularly for ARIA-H), which exceeded 80%. This large span of heterogeneity in imaging outcomes might be due to variations in imaging techniques, PET tracers used, pre-morbid level of amyloid in the brain, disease stage, dosage and duration of treatment, or the definition of amyloid negativity. Moreover, the discrepancies in ARIA outcomes could be related to variability in ApoE4 status, cerebrovascular risk in treatment-naive patients, differences in ARIA definitions, differences in surveillance, as well as differences in analyzed populations between trials and real-life patients.

Regarding the comparative efficacy and safety of the two approved AAAs, lecanemab and donanemab, there are few available data. They suggest that donanemab had a slightly more favorable profile on cognitive scales (CDR-SB, ADCS-ADL, ADAS-cog) compared to lecanemab and that it significantly outperformed symptomatic drugs [38], while lecanemab exhibited the highest efficiency on the ADCS-ADL [35]. Concerning safety, lecanemab exhibited a lower risk of ARIA-E and ARIA-H than donanemab [35,37,88]. However, caution is needed when making direct comparisons between the two antibodies, as there were variations in patient selection, amyloid and tau eligibility criteria, ApoEε4 distribution, dosing, stopping rules, follow-up, and MRI surveillance. On the other hand, donanemab had a slightly favorable profile compared to lecanemab in terms of overall adverse events [35,38]. In terms of patient convenience, another potential advantage of donanemab is monthly intravenous infusions compared to the 2-week infusions of lecanemab. At this point, it should be recognized that the two pivotal phase III trials, CLARITY-AD (lecanemab) and TRAILBLAZER-ALZ2 (donanemab), employed different biomarker and clinical inclusion and exclusion criteria, resulting in subtle but potentially meaningful differences in patient cohorts. Specifically, patients in TRAILBLAZER-ALZ2 were clinically and biologically more advanced than those in CLARITY-AD [10,11]. Another issue is the pharmacodynamic differences between lecanemab and donanemab, as lecanemab targets soluble protofibrillar forms of Aβ, which are not visible on amyloid PET scans and may have their own toxicity [95]. Direct comparisons of safety and efficacy data between the two trials are not valid, and only associations can be made at this point. Another difference is the limited duration of donanemab treatment, compared to the open-label extension of lecanemab treatment. Moreover, the issue of anti-drug antibodies (ADAs) or “neutralizing antibodies” for AAAs and whether this might contribute to the effectiveness of lecanemab or donanemab is not so well-studied. These antibodies can interfere with the pharmacodynamic interaction between the therapeutic protein and its target [96]. In pivotal trials, donanemab-treated participants had more frequent treatment-emergent ADAs than lecanemab, with the latter exhibiting generally low titers [97,98]. While these antibodies increased the clearance of donanemab, they did not affect the reduction of plaques or clinical efficacy, as measured by the CDR-SB [97]. The immunogenicity of lecanemab has not been sufficiently evaluated due to the limitations of the ADA assay; however, lecanemab is a low-risk molecule for immunogenicity [99]. Therefore, this umbrella review does not lead to a straightforward conclusion of superiority of one antibody over the other.

**Figure 7:**
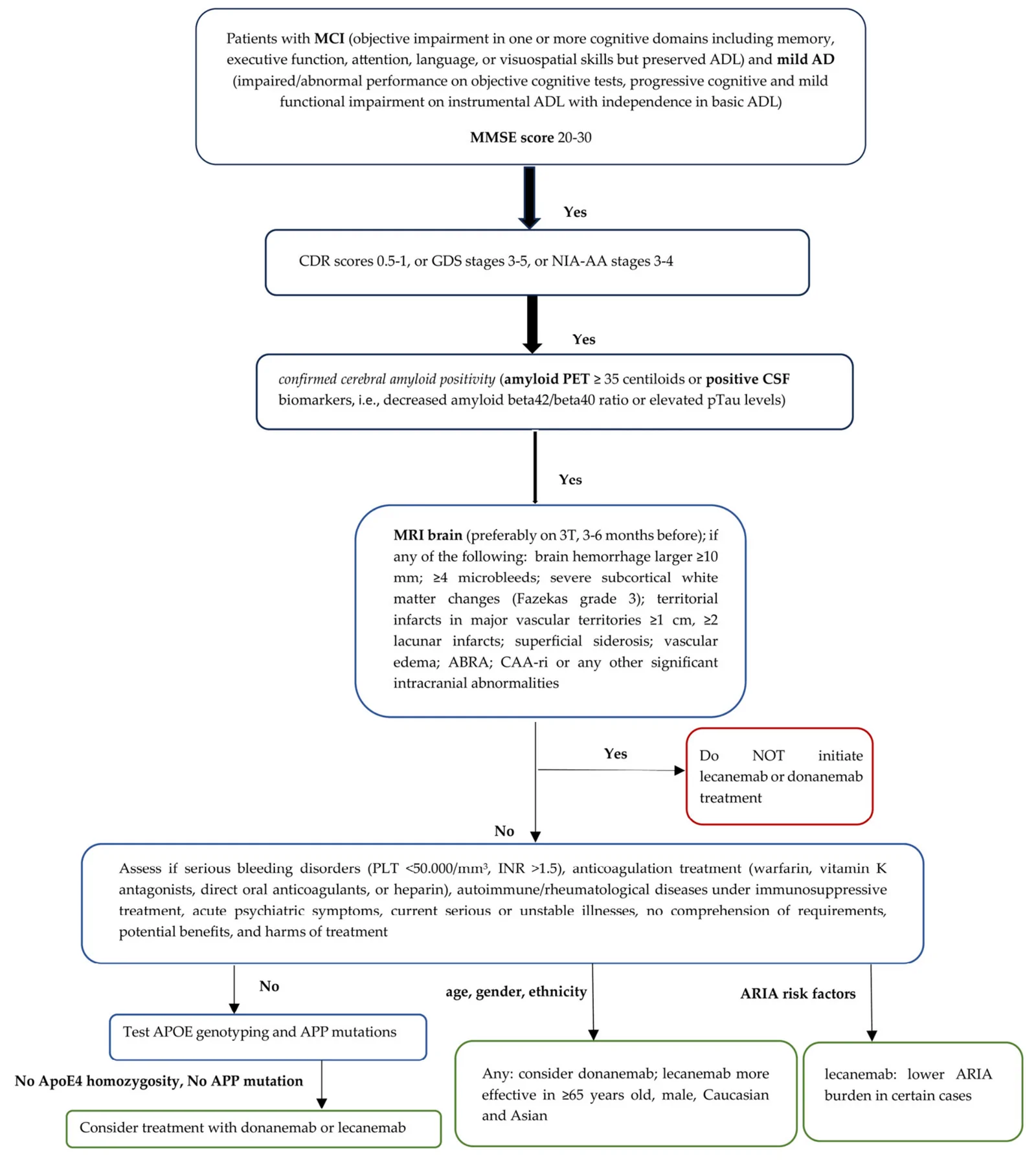
Workflow for choosing the appropriate candidates with AD for treatment initiation with AAAs. Abbreviations: ADL: activities of daily living, CDR: clinical dementia rating, GDS: Global Deterioration Scale, NIA-AA: National Institute on Aging-Alzheimer’s Association, PET: positron emission tomography, CSF: cerebrospinal fluid, pTau: phosphorylated Tau, PLT: platelets, ABRA: Aβ-related vasculitis, CAA-ri: CAA-related inflammation.

**Figure 8:**
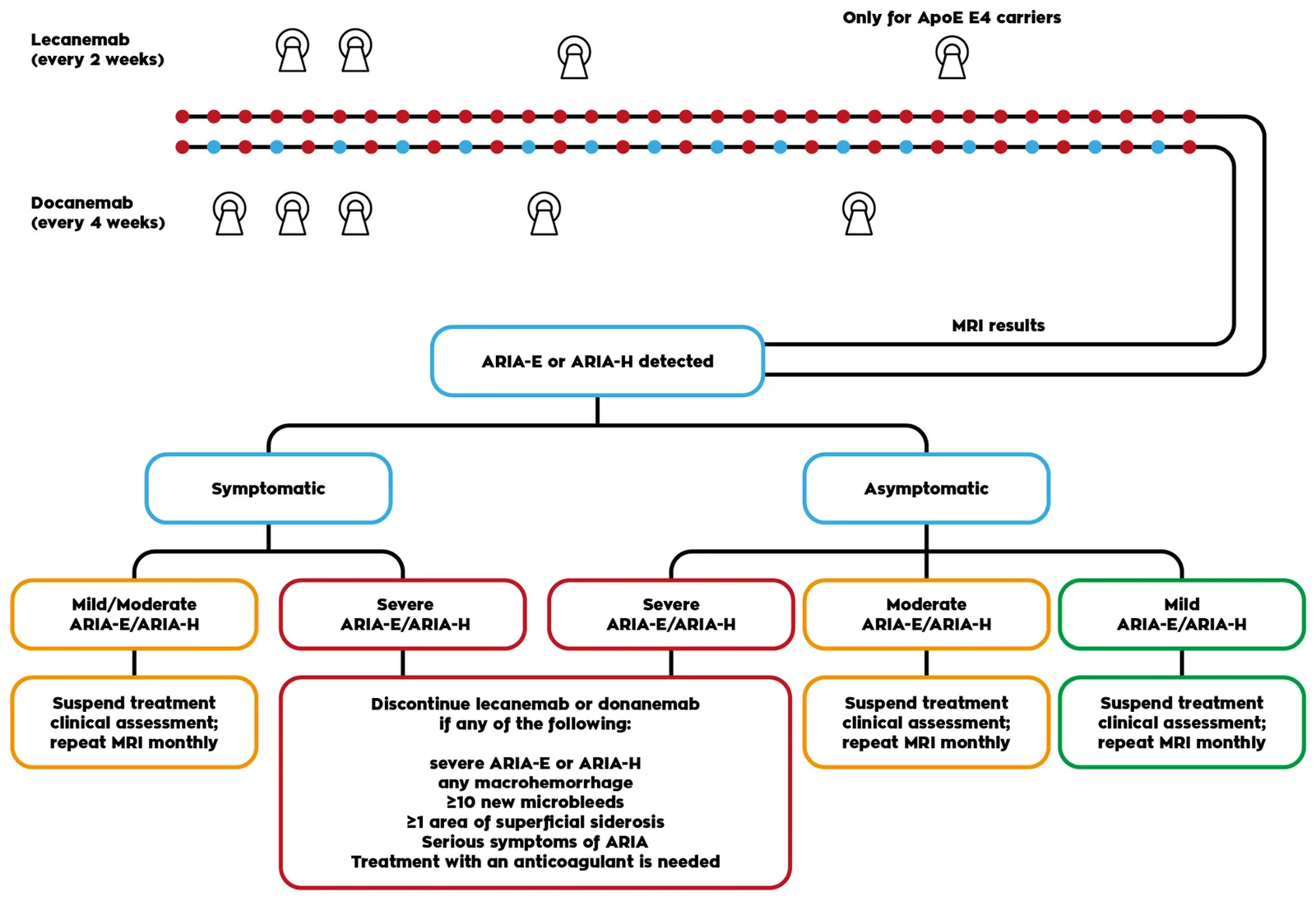
Workflow on follow-up of patients on AAA treatment. Each red dot represents the timing of infusion, while MRI follow-ups are depicted with a drawing of MRI device. Abbreviations: MRI: magnetic resonance imaging, ARIA: amyloid-related imaging abnormalities, ARIA-H: amyloid-related imaging abnormalities-hemorrhage/hemosiderosis, ARIA-E: amyloid-related imaging abnormalities-edema.

The above findings have implications for cautious implementation in clinical and policy settings. Notably, the introduction of such therapies into clinical routine presents several practical challenges. Critical components comprise careful candidate selection, ARIA risk stratification, infusion workflows, monitoring protocols, and adverse event management strategies (see Figure 7 and Figure 8 for practical workflows on selection and follow-up of appropriate candidates). The selection of patients should take into account age, disease stage, confirmation of amyloid, comorbidities such as arterial hypertension and pre-existing cerebral microhemorrhages, ApoE4 status, and the ability to keep up with regular MRI monitoring. Furthermore, there are problems with implementation in clinical practice, such as differences in the community real-world population compared to those enrolled in clinical trials. This refers to ApoE4 carrier status and variations in treatment administration and setting, such as the presence of experienced professionals in clinical trials compared to community clinicians and the limited access to imaging such as amyloid PET scan [100]. Another issue is the diversity of protocols in conducting PET scans in radiological centers worldwide. PET scans should be performed using an FDA-approved Aβ radiotracer (18F-florbetaben, 18F-florbetapir, 18F-flutametamol) [22]. Although there are FDA-approved software packages that enable centiloid quantification of Aβ-PET, these are not yet routinely used in clinical practice. From a pragmatic point of view, financial coverage of multiple amyloid PET scans is currently unclear, and scan interpretation in clinical practice does not routinely include the quantification needed to discontinue treatment as in clinical trials [22]. There are also gaps in our understanding of the optimal duration of AAA treatment [22].

Concerning patient diversity around the world, since there are differences in the frequency of amyloid positivity among various ethnicities (e.g., less common in Asian and African-American compared to Caucasian individuals) [19] and the trials included different proportions of these ethnicities, a factor that should be considered is that there may be ethnic and racial differences in treatment responses, therapy preferences, adverse events, treatment adherence, and patient–caregiver relationships. At the moment, there is limited experience with early-age-of-onset AD (symptom onset at age below 65 years), more severe AD dementia or atypical AD forms including logopenic variant, primary progressive aphasia, posterior cortical atrophy, or behavioral or dysexecutive AD, as patients included in trials had relatively typical amnestic AD phenotypes [19,22]. There is also limited experience with patients with disease-causing mutations in the amyloid precursor protein (APP), presenilin-1 (PSEN1), and presenilin-2 (PSEN2) genes [22]. There are also practical considerations for clinicians in the acute setting. As ARIA can resemble stroke symptoms, a “stroke protocol” with head CT/CT angiogram is advised to be conducted. However, the “stroke protocol” may fail to detect even advanced ARIA [22]. Conversely, severe cases of ARIA-E can be associated with punctate regions of restricted diffusion on DWI consistent with acute ischemia, but these are typically much smaller [22]. Moreover, patients on AAAs should not be treated with acute thrombolytics (intravenous or intra-arterial) until safety evidence of their combined use is available [19,22]. Patients with large vessel occlusion can be treated with intravascular thrombectomy [22].

Overall, the modest efficacy of AAAs, in terms of the slowing of cognitive decline, should be weighed against the significant safety burden, and particularly the ARIA risk. Importantly, although the clinical benefits on cognitive scales such as the CDR-SB and ADAS-Cog are statistically significant, they are often not clinically relevant. This implies that patients or caregivers will probably not perceive any difference in everyday cognitive functioning. Moreover, the long-term impact of these marginal cognitive benefits on disease progression remains uncertain [92]. It is also unclear if the robust effect of AAAs on amyloid PET translates into clinical benefits [101]. Moreover, AAAs clearly increase the risk of angiogenic edema and microhemorrhages, i.e., ARIA risk. More specifically, the relative increase in risk was greater for ARIA-E compared to ARIA-H for both AAAs (approximately 8–12 times compared to 2–6 times higher than placebo, respectively). These adverse effects appear to be, in most cases, mild, dose-dependent, self-limiting, and, in the majority of patients, related to the presence of the ApoEε4 gene. However, they require intensified MRI monitoring, dose adjustments, or temporary treatment interruptions. Importantly, a few fatal cases have been connected to ARIA. Infusion-related reactions after the administration of AAAs are also an issue; however, these were most commonly mild or moderate [21,93]. Therefore, the benefit–risk ratio should be weighed before the clinical decision of starting or continuing a treatment with AAAs is reached. Their unfavorable profile, at least based on the current evidence, limits clinical applicability in patients with AD who are usually frail, not only as a result of the disease but also due to age and/or various comorbidities. Therefore, the results do not support a robust clinical rationale for the widespread adoption of these therapies and raise doubts about the validity of the amyloid clearance paradigm as a disease-modifying strategy in AD.

At this point, it should be stressed that AD has a multifactorial nature. The amyloid cascade hypothesis [102], as the etiological underpinning of AD, states that the accumulation of β-amyloid precedes and leads to cognitive decline. Traditional models centered on amyloid-β and tau pathology have expanded to encompass processes such as neuroinflammation, synaptic dysfunction, and gut–brain axis disruption [103]. Indeed, the natural history of AD progression does not correlate with brain amyloid formation [104]. Aβ-deposition is not related to the severity of AD, as mentally healthy individuals can have amyloid plaques in the brain, and tau pathology appears to precede Aβ deposits in patients younger than 30 years with AD [105]. Therefore, there is a growing body of research that the amyloid theory as the etiology of AD needs to be re-evaluated [77,104,106]. It is possible that Aβ-deposition is a secondary phenomenon following another initial injury or the brain’s response to neuronal destruction [107]. More specifically, many other theories of the pathophysiology of AD have been proposed, such as the disrupted glutamate signaling hypothesis and “excitotoxicity”, which can impair synaptic structure and function, leading to synapse loss and ultimately neuron cell death [104]. Moreover, the presence of chronic neuroinflammation, breakdown of the blood–brain barrier, and increased levels of inflammatory mediators play a central role in the pathogenesis of AD [108,109]. Chronic microglial activation due to the aggregation of tau protein and amyloid peptides, as well as abnormal neuron–microglia interactions, can lead to irreversible neuron damage [108]. While microglia, the resident immune cells of the CNS, play a crucial role in AD, recent evidence suggests the infiltration of cerebral parenchyma by peripheral immune cells, including T- and B-lymphocytes, neutrophils, NK cells, and monocytes, in AD [108,109]. From another point of view, the dysfunction of the glymphatic system, which may contribute to the reduced clearance of the amyloid and tau burden from the brain, is still a matter of active research [108,110]. The newly formed “triple-hip hypothesis” offers another interesting perspective, in which Aβ-deposition is a downstream event and a result of the sequential interaction of early blood–brain barrier breakdown, entry or reactivation of pathogens within the brain, and maladaptive innate immune responses that cause chronic neuroinflammation [111]. Thus, it seems that Aβ is an invariable accompaniment of the disease process in AD; however, it neither drives nor mitigates the disease process itself [77].

### Limitations

This umbrella review suffers from certain limitations. One of the most important attributes of the evidence included is that most clinically meaningful conclusions rely on a few, large, phase II or phase III trials. Lecanemab had pivotal trial data in CLARITY-AD and evidence from Study 201, a phase IIb trial [10]. The central trials for donanemab were TRAILBLAZER-ALZ [51] and TRAILBLAZER-ALZ 2 [11]. Thus, the findings of the umbrella review should be interpreted as a descriptive analysis of the interpretation of these large trials in previous meta-analyses rather than as a new pooled analysis of all the individual patient data. The lack of independence between the included reviews needs to be taken into account when assessing the strength of evidence. The corrected covered area was 13.04%, which means that there was high overlap of the primary pivotal trials. Using the results of a primary study multiple times in the same analysis overstates the sample size and number of events, falsely leading to greater precision in the analysis [112]. This overlap reduces the independence of data and can create a false sense of high certainty through repeated reinforcement of the same underlying information. Notably, there is currently no standard methodological approach to deal with overlap in primary studies across reviews [112]. The umbrella review, therefore, finds a high degree of agreement among meta-analyses; this, however, is in part due to the inclusion of the same underlying studies in repeated analyses. Regarding heterogeneity, there was considerable variation ranging from negligible to very high degrees of heterogeneity. Most clinical outcomes had low to moderate heterogeneity, while the imaging outcomes and other safety outcomes were more heterogeneous. Due to the significant heterogeneity for safety, especially for ARIA-H, care should be taken when interpreting pooled safety estimates. The observed rates of ARIA may be dramatically affected by differences in ApoE4 status, MRI monitoring, baseline cerebrovascular risk, and real-world eligibility.

The results of this umbrella review are mainly based on trial data. Real-world patients tend to differ from those selected to participate in a clinical trial. Another issue is that the existing RCTs were conducted in patients with mild or early AD and possibly fewer comorbidities than real-world patients. Real-world patients with early-stage AD tend to be older, more diverse and less well educated and have greater rates of comorbidities and higher concomitant medication use than clinical trial participants. Healthcare systems are also less well-resourced and coordinated, compared to clinical trial programs [113]. Moreover, the duration of clinical trials was relatively short to assess safety and, foremost, long-term efficacy, ranging between 18 and 21 months for donanemab and between 6 and 24 months for lecanemab. Lastly, there are insufficient data on the results of the intervention at a later stage or, conversely, at a very early stage, ideally at a preclinical stage, i.e., up to 10 years before disease onset. Recent trials such as TRAILBLAZER-ALZ 3 (a phase 3 prevention trial) [114] and TRAILBLAZER-ALZ 5 (phase 3 safety and efficacy trial, NCT05508789 in ClinicalTrials.gov), which investigate the efficacy of donanemab in patients at high risk of developing AD and prodromal AD, respectively, aim to address these issues. The results of the AHEAD 3–45 trial are also expected to determine whether lecanemab therapy can slow Aβ and tau accumulation and prevent cognitive decline during its preclinical stage [115].

## 5. Conclusions

In this paper, a comprehensive approach to the treatment with monoclonal antibodies in AD was attempted, with the analysis of high-quality systematic reviews and meta-analyses published in the last decade to obtain safe and objective conclusions for physicians. The use of AAAs was a very promising concept in view of the lack of effective causative therapies for AD. Overall, the results confirm a disease-modifying effect of lecanemab and donanemab in MCI and early AD, supported by clinical and imaging effects. This umbrella review does not, however, lead to a straightforward conclusion of superiority of one antibody over the other. Importantly, although the clinical benefits are statistically important, they are often not clinically meaningful. There are also insufficient data regarding the effect of these drugs on quality of life or caregiver burden. Moreover, AAAs clearly increase ARIA risk, which appears to be, in most cases, mild, self-limiting, and, in the majority of patients, related to ApoEε4 gene presence. However, a few fatal cases have been associated with ARIA, and therefore, the benefit–risk ratio should be weighed before reaching the clinical decision of starting or continuing a treatment with AAAs. Whether the net benefit is worth the net harm depends on patient factors, baseline risk, and monitoring ability. These drugs, therefore, should be regarded as agents that can slow down the cognitive decline in well-selected patients rather than as restorative drugs. Clinicians must set realistic expectations for patients and their caregivers, as long-term outcomes beyond three years remain currently under investigation. Patients and their caregivers must understand the potential benefits and harms of the treatment, the occurrence of ARIA, and the need to stick to a very strict administration and follow-up protocol. Patients and caregivers should be aware that anti-amyloid treatment is not expected to arrest disease progression or prevent the progression of symptoms over the long term, as well as eventual functional decline. Since most clinicians have limited experience with AAA use, education regarding the evaluation and differential diagnosis of early-stage cognitive–behavioral impairment, access to AD CSF or PET biomarker testing and APOE genotyping, and the ability to interpret the clinical, biomarker, and genetic results across a wide array of clinical scenarios are foundational for the success of AAA treatment. The possibility of critical adverse events such as large intracranial hemorrhage as a result of ARIA exerts considerable pressure on clinicians, and exclusion criteria for receiving an AAA often resemble the stroke protocol for thrombolysis. Moreover, AAA treatment requires access to infusion centers or AD-specific infusion resources, which are not available at all neurology centers in various countries.

The results of this umbrella review can be used by clinicians and trialists to inform the design of therapeutic interventions through new randomized clinical trials. Further research is needed to assess the efficacy, safety, and maintenance of treatment effects of lecanemab and donanemab in comparison to established therapies. Longer trials (e.g., longer than 3 years) and real-world data may be necessary to see these effects grow into clinically meaningful changes. Therefore, these new treatments can only be regarded as the first step in a new era and a conceptual shift from a syndrome- and care-based understanding of AD to biologically defined AD with molecular-targeted causal treatment. Patient-centered, supported, and shared decision-making processes are warranted. The foundational medical concept “to do good and do no harm”, which is deeply tied to Hippocrates [116], remains as relevant today as it was 2500 years ago.

## Figures and Tables

**Table 1:** Metric-separated review-level ADAS-Cog evidence map for lecanemab and donanemab versus placebo.

Review/Antibody	Outcome Version	Metric	Reported Estimate (95% CI)	Direction
Qiao et al. (2024) [35]/Lecanemab	ADAS-Cog	MD	−1.09 (−1.69, −0.49)	Favors Treatment
Qiao et al. (2024) [35]/Donanemab	ADAS-Cog	MD	−1.78 (−3.52, −0.01)	Favors Treatment
Qiao et al. 2023 [36]/Lecanemab	ADAS Cog14	WMD	−1.11 (−1.64, −0.57)	Favors Treatment
Lyu et al. (2023) [33]/Lecanemab	ADAS-Cog	SMD	−0.17 (−0.26, −0.07)	Favors Treatment
Lyu et al. (2023) [33]/Donanemab	ADAS Cog	SMD	−0.35 (−0.58, −0.01)	Favors Treatment
Su et al. (2025) [38]/Lecanemab	ADAS-Cog	SMD	−0.51 (−0.71 to −0.31)	Favors treatment
Su et al. (2025) [38]/Donanemab	ADAS-Cog	SMD	−0.57 (−0.79 to −0.36)	Favors treatment

Abbreviations: ADAS-Cog: Alzheimer’s Disease Assessment Scale–Cognitive Subscale, MD: mean difference, WMD: weighted mean difference, SMD: standardized mean difference.

**Table 2:** Metric-specific review-level imaging estimates for lecanemab and donanemab versus placebo.

Review Paper	AAA	PET Measure	Mean Difference	95% CI
Qiao et al. (2023) [36]	Lecanemab	PET amyloid load	WMD −35.44	95% CI −65.22 to −5.67
Qiao et al. (2023) [36]	Lecanemab	SUVr	WMD −0.15	95% CI −0.48 to 0.19
Qiao et al. (2024) [35]	Donanemab	SUVr	MD −0.37	95% CI −0.57 to −0.17

Abbreviations: AAA: anti-amyloid antibody, SUVr: standardized uptake value ratio, PET: positron emission tomography, WMD: weighted mean difference, MD: mean difference.

**Table 3:** Published review-level ARIA-E and ARIA-H relative-effect estimates for lecanemab and donanemab versus placebo.

Review	Antibody	Outcome	Effect Measure	Estimate	95% CI
Qiao et al. (2024) [35]	Lecanemab	ARIA-E	OR	8.32	5.50–13.15
Qiao et al. (2024) [35]	Donanemab	ARIA-E	OR	12.26	6.58–25.19
Qiao et al. (2023) [36]	Lecanemab	ARIA-E	OR	8.95	5.36–14.95
Qiao et al. (2023) [36]	Lecanemab	ARIA-H	OR	2.00	1.53–2.62
Qiao et al. (2024) [35]	Lecanemab	ARIA-H	OR	2.12	1.60–2.83
Qiao et al. (2024) [35]	Donanemab	ARIA-H	OR	5.77	3.39–10.26

Abbreviations: ARIA: amyloid-related imaging abnormalities, ARIA-E: amyloid-related imaging abnormalities with edema, ARIA-H: amyloid-related imaging abnormalities-hemorrhage, OR: odds ratio.

## Data Availability

The original contributions presented in this study are included in the article. Further inquiries can be directed to the corresponding author.

## Supplementary Materials

The following supporting information can be downloaded at: https://www.mdpi.com/article/10.3390/jcm15176782/s1, Supplementary Table S1. Systematic reviews and meta-analyses of lecanemab and donanemab included in the umbrella review [117]. Supplementary Table S2. AMSTAR 2 rating for included studies. Supplementary Table S3. AMSTAR 2 rating for excluded studies [74,87,90,92,94,101,118,119,120,121,122,123,124,125,126,127,128,129,130,131,132,133,134,135,136,137,138]. File S1. PRISMA 2020 Checklist. File S2. PRISMA 2020 for Abstracts Checklist.
