# Supplementary material for: Anti-Amyloid Antibodies in the Treatment of Alzheimer’s Disease: An Umbrella Review
Source: J Clin Med. 2026 Sep 1;15(17):6782. doi: 10.3390/jcm15176782 (PMC13566784; doi:10.3390/jcm15176782)
Supplement: Supplementary file 1 [file jcm-15-06782-s001.zip › File S2. PRISMA 2020 for Abstracts Checklist.pdf]

PRISMA

PRISMA 2020 for Abstracts Checklist

Section and Topic	Item #	Checklist item	Reported (Yes/No)
**TITLE**			
Title	1	Identify the report as a systematic review.	Yes, line 3
**BACKGROUND**			
Objectives	2	Provide an explicit statement of the main objective(s) or question(s) the review addresses.	Yes, Lines 17-19
**METHODS**			
Eligibility criteria	3	Specify the inclusion and exclusion criteria for the review.	Yes, Lines 19-22
Information sources	4	Specify the information sources (e.g. databases, registers) used to identify studies and the date when each was last searched.	Yes, Lines 19-20
Risk of bias	5	Specify the methods used to assess risk of bias in the included studies.	Yes, line 20
Synthesis of results	6	Specify the methods used to present and synthesise results.	Yes, line 22-23
**RESULTS**			
Included studies	7	Give the total number of included studies and participants and summarise relevant characteristics of studies.	Yes, line 23
Synthesis of results	8	Present results for main outcomes, preferably indicating the number of included studies and participants for each. If meta-analysis was done, report the summary estimate and confidence/credible interval. If comparing groups, indicate the direction of the effect (i.e. which group is favoured).	Yes, lines 23-31
**DISCUSSION**			
Limitations of evidence	9	Provide a brief summary of the limitations of the evidence included in the review (e.g. study risk of bias, inconsistency and imprecision).	No (in the main text)
Interpretation	10	Provide a general interpretation of the results and important implications.	Yes, 32-35
**OTHER**			
Funding	11	Specify the primary source of funding for the review.	No (in the main text)
Registration	12	Provide the register name and registration number.	No (in the main text)

From: Page MJ, McKenzie JE, Bossuyt PM, Boutron I, Hoffmann TC, Mulrow CD, et al. The PRISMA 2020 statement: an updated guideline for reporting systematic reviews. BMJ 2021;372:n71. doi: 10.1136/bmj.n71. This work is licensed under CC BY 4.0. To view a copy of this license, visit

PRISMA

PRISMA 2020 for Abstracts Checklist

https://creativecommons.org/licenses/by/4.0/
