# Supplementary material for: Anti-Amyloid Antibodies in the Treatment of Alzheimer’s Disease: An Umbrella Review
Source: J Clin Med. 2026 Sep 1;15(17):6782. doi: 10.3390/jcm15176782 (PMC13566784; doi:10.3390/jcm15176782)
Supplement: Supplementary file 1 [file jcm-15-06782-s001.zip › supplementary tables.pdf]

No.	Author, year (review/meta-analysis)	Studies included	Studied AAA, dose and duration of treatment	Number of included patients	Main conclusions on clinical efficacy of AAA (vs placebo)	Main conclusions on imaging efficacy of AAA (vs placebo)	Main conclusions on safety of AAA (vs placebo)	Quality (AMSTAR 2)	Heterogeneity and risk of bias
1	Wang et al. (2025) [31] Meta-analysis	Mintun et al. (2021) [51]; Van Dyck et al. (2023) [10]; McDade et al. (2022) [61]; Swanson et al. (2021) [12]; Honig et al. (2024) [59].	Donanemab: 700 mg for three doses, then 1,400 mg. Lecanemab: 10 mg/kg every 2 weeks; phase II regimens also included. Duration: donanemab up to 72 weeks; lecanemab 18–24 months.	Donanemab: 131 vs 126 placebo. Lecanemab: 2,620 vs 2,288 placebo.	Mintun: no statistically significant change in MMSE, iADRS or CDR-SB at 76 weeks. CLARITY-AD: significant benefit with lecanemab on CDR-SB, ADAS-Cog14, ADCOMS and ADCS-MCI-ADL ($p<0.001$).	Significant amyloid reduction with donanemab and lecanemab on PET. McDade: 81% of lecanemab recipients converted to amyloid-negative status at 18 months.	ARIA-E increased (RR 7.52–33.66); ARIA-H increased (RR 1.95–2.02). Symptomatic ARIA-E increased; no clear difference in death or other serious adverse events.	Moderate (13/16)	Clinical: low–moderate heterogeneity. Imaging: high (PET I^2=95%). Selected ARIA analyses: I^2=0%; total ARIA: I^2=91%. RoB: low.
2	Zeng et al. (2024) [32] Network meta-analysis	Van Dyck et al. (2023) [10]; Sims et al. (2023) [11]; Berry et al. (2023) [55]; Mintun et al. (2021) [51].	Donanemab: 700 mg ×3, then 1,400 mg monthly. Lecanemab: 10 mg/kg every 2 weeks plus phase II regimens. Duration: 18 months.	Donanemab: 991 vs 1,002 placebo. Lecanemab: 1,446 vs 1,113 placebo.	Both agents favored treatment for subjective memory decline: donanemab −0.239; lecanemab −0.194 (both $p<0.001$; I^2=0%).	Not reported separately.	ARIA-E: 12.6% lecanemab; 24.0% donanemab. ARIA-H: 17.3% lecanemab; 19.7% donanemab. No significant increase in serious AEs or mortality.	Moderate (14/16)	Clinical I^2=0%. AE heterogeneity high (65.0–81.6%); serious AEs and mortality low. RoB: low (RoB 2).
3	Lyu et al. (2023) [33] Meta-analysis	Mintun et al. (2021) [51]; Swanson et al. (2021) [12]; Van Dyck et al. (2023) [10].	Donanemab: 700 mg titration to 1,400 mg monthly. Lecanemab: 2.5–10 mg/kg in phase II; 10 mg/kg every 2 weeks in phase III. Duration: 18 months.	Donanemab: 131 vs 126 placebo. Lecanemab: 1,446 vs 1,113 placebo.	Lecanemab favored treatment for ADAS-Cog (SMD −0.17) and CDR-SOB (SMD −0.19). Donanemab favored ADAS-Cog (SMD	Not reported separately.	Not reported separately for lecanemab and donanemab.	Moderate (13/16)	ADAS-Cog: low heterogeneity. CDR-SOB: moderate heterogeneity (I^2 43.2–50.9%). RoB: low (RoB 2).

					−0.35); CDR-SOB did not exclude the null.				
4	Qi et al. (2026) [34 Meta-analysis	Lecanemab RCTs and real-world evidence: Swanson et al. (2021) [12], Van Dyck et al. (2023), Shields et al. (2024) [10], Bregman et al. (2025) [65], Paczynski et al. (2025) [66], Rosenbloom et al. (2025) [67] reports.	Lecanemab 10 mg/kg every 2 weeks.<br>Duration: 15–72 weeks.	N=1,576 (safety-focused analysis).	Clinical efficacy not assessed.	Not assessed.	Overall ARIA: 19%; ARIA-E: 10%; ARIA-H: 11%.<br>Symptomatic ARIA: 3%.<br>ARIA incidence increased with APOE ε4 gene dose; discontinuation due to ARIA: 5%.	High (16/16)	ARIA I^2=42.3%; ARIA-E I^2=54.4%; ARIA-H I^2=86.2%.<br>RoB: low (RoB 2 for RCTs; JBI for RWE).
5	Qiao et al. (2024) [35] Comparative network meta-analysis	Lowe et al. (2021) [50]; Mintun et al. (2021) [51]; Shcherbinin et al. (2022) [54]; Logovinsky et al. (2016) [60]; Swanson et al. (2021) [12]; Van Dyck et al. (2023) [10].	Donanemab: 700 mg titration to 1,400 mg monthly.<br>Lecanemab: phase I–III doses, including 10 mg/kg every 2 weeks.<br>Duration: donanemab 72–84 weeks; lecanemab 6–18 months.	Donanemab: 292 vs 261 control.<br>Lecanemab: 1,506 vs 1,133 control.	Donanemab had the largest ADAS-Cog benefit (MD −1.78).<br>Lecanemab improved CDR-SB (MD −0.34), ADAS-Cog (MD −1.09) and ADCS-ADL (MD +2.00).	Donanemab improved amyloid PET SUVr (MD −0.37).<br>Lecanemab SUVr estimate did not exclude the null.	Lecanemab AEs: OR 2.16.<br>ARIA-E: OR 12.26 donanemab; OR 8.32 lecanemab.<br>ARIA-H: OR 5.77 donanemab; OR 2.12 lecanemab.	High (15/16)	Random-effects models used when I^2>50%.<br>RoB: low–moderate.
6	Qiao et al. (2023) [36] Meta-analysis	Logovinsky et al. (2016) [60]; Swanson et al. (2021) [12];	Lecanemab dose-escalation/maintenance regimens, including 10 mg/kg every 2 weeks.	Lecanemab: 1,695 vs 1,413 placebo.	Lecanemab improved CDR-SB (WMD −0.45), ADCOMS (WMD	SUVr did not differ significantly.	No significant difference in overall AEs.	High (15/16)	CDR-SB/ADCOMS/ARIA: I^2=0%.

		McDade et al. (2022) [61]; Van Dyck et al. (2023) [10].	Duration: 3–24 months.		−0.05) and ADAS-Cog14 (WMD −1.11).	PET amyloid load favoured lecanemab (WMD −35.44), but heterogeneity was high.	ARIA-E OR 8.95; ARIA-H OR 2.00.		SUVr I^2=97%; PET amyloid I^2=98%; AEs I^2=92%.<br>RoB: low.
7	Terao & Kodama (2024) [40]<br>Network meta-analysis	Mintun et al. (2021) [51]; Sims et al. (2023) [11]; Swanson et al. (2021) [12]; Van Dyck et al. (2023) [10].	Donanemab: 1,400 mg monthly.<br>Lecanemab: 2.5–10 mg/kg every 2 or 4 weeks.<br>Duration: 18 months.	Donanemab: 991 vs 1,002 control.<br>Lecanemab: 1,011 vs 1,113 control.	No statistically significant difference in cognitive function relative to the broader network of comparator interventions.	Not reported.	Donanemab and lecanemab were less well tolerated than placebo.	High (16/16)	No significant heterogeneity reported for cognitive change or tolerability.<br>RoB: mostly low; some concerns in Mintun and Swanson.
8	Dantas et al. (2024) [41]<br>Systematic review and meta-analysis	Swanson et al. (2021) [12]; Van Dyck et al. (2023) [10]; Mintun et al. (2021) [51].	Donanemab: 700–1,400 mg monthly.<br>Lecanemab: phase II regimens and 10 mg/kg every 2 weeks.<br>Duration: 18 months.	Donanemab: 131 vs 126 placebo.<br>Lecanemab: 1,446 vs 1,113 placebo.	Class-level evidence: modest benefit on CDR-SB, ADAS-Cog, ADCS-ADL and MMSE.<br>Drug-specific findings varied by disease stage and outcome.	Not reported.	Class-level ARIA-H risk increased (RR 1.80).<br>ARIA-E increased; symptomatic/serious ARIA increased.	High (15/16)	Clinical outcomes low–moderate heterogeneity.<br>ARIA-E I^2=10%; ARIA-H I^2=87%.<br>RoB: low.
9	Shim et al. (2026) [37]<br>Systematic review and meta-analysis	Lecanemab and donanemab pivotal trials and reports, including Van Dyck et al. (2023) [10]; Swanson et al. (2021) [12]; Honig et al. (2024) [59];	Donanemab: 700 mg ×3, then 1,400 mg monthly.<br>Lecanemab: 10 mg/kg every 2 weeks; phase II regimens.<br>Mostly 18 months.	Donanemab: 2,414 vs 2,868 placebo.<br>Lecanemab: 5,918 vs 4,641 placebo.	Reported subgroup-specific cognitive effects; donanemab generally favoured across age groups.<br>Lecanemab effects varied by sex, age and APOE ε4 status.	Not reported.	Pooled ARIA-E RR: 13.08 donanemab; 7.96 lecanemab.<br>Pooled ARIA-H RR: 2.65 donanemab; 1.84 lecanemab.	High (15/16; 1 N/A)	ADE heterogeneity high; SAE heterogeneity lower.<br>RoB: 15/16 studies low risk; one with some concerns.

		Mintun et al. (2021) [51]; Sims et al. (2023) [11]; Shcherbinin et al. (2022) [54]; Sato et al. 2024 [53]; Zimmer et al. 2025 [62]; Salloway et al. 2025 [52]; Wang, Serap Monkul Nery et al. 2025 [64].							
10	Su et al. (2025) [38] Network meta-analysis	Sims et al. (2023) [11]; Mintun et al. (2021) [51]; Swanson et al. (2021) [12].	Donanemab: 700 mg ×3, then 1,400 mg every 4 weeks. Lecanemab: 2.5–10 mg/kg every 2 or 4 weeks. Duration: 18 months.	Donanemab: 991 vs 1,002 placebo. Lecanemab: 587 vs 238 placebo.	Network findings varied by comparator and outcome; active treatment generally favored for selected cognitive/functional outcomes, but some direct placebo CDR-SB estimates crossed the null.	Not reported.	No significant difference in serious AEs versus placebo for either antibody in direct comparisons.	High (16/16)	Heterogeneity cited as a limitation. RoB: mostly low; ~25–30% of comparisons had some concerns.
11	Wei & Li (2025)[39] Systematic review and meta-analysis	Van Dyck et al. (2023) [10]; Sims et al. (2023) [11].	Lecanemab: 10 mg/kg every 2 weeks. Donanemab: 700–1,400 mg. Duration: 18 months.	Donanemab: 860 vs 876 placebo. Lecanemab: 859 vs 875 placebo.	Donanemab CDR-SB: −0.70; lecanemab CDR-SB: −0.45. Donanemab MMSE favoured treatment.	Not reported.	ARIA-E increased with lecanemab.	High (16/16)	Selected CDR-SB and ARIA-E analyses had I^2=0%. RoB generally low; some concerns for Van Dyck.

Abbreviations: MMSE: Mini-Mental Status Examination, ADAS-Cog: Alzheimer's Disease Assessment Scale–Cognitive Subscale, CDR-SOB: Clinical Dementia Rating Sum of Boxes, iADRS: Integrated Alzheimer's Disease Rating Scale, ADCOMS: Alzheimer's Disease Composite Score, ADCS-ADL: Alzheimer's Disease Cooperative Study - Activities of Daily Living, SCD: Subjective Cognitive Decline, SMD: Subjective Memory Decline, SUVR/SUVr: Standardized Uptake Value Ratio in amyloid PET, ARIA: Amyloid-Related Imaging Abnormalities, ARIA-E: ARIA-oedema/effusion, ARIA-H: hemosiderosis/microhemorrhages

Supplementary Table S1. Systematic reviews and meta-analyses of lecanemab and donanemab included in the umbrella review

Author (year)	Q1	**Q2**	Q3	**Q4**	Q5	Q6	**Q7**	Q8	**Q9**	Q10	**Q11**	Q12	**Q13**	Q14	**Q15**	Q16	Rating
Dantas (2024)(Dantas et al., 2024) [41]	Yes	Yes	Yes	Yes	Yes	Yes	Yes	Yes	Yes	No	Yes	Yes	Yes	Yes	Partial Yes	Yes	15/16 High
Lyu (2023)(Lyu, Lyu, Huang, & Fang, 2023) [33]	Yes	Yes	No	Yes	Yes	Yes	Partial yes	Yes	Yes	No	Yes	Yes	Yes	Yes	Yes	Yes	14/16 Moderate
Qi (2026)(Qi et al., 2026) [34]	Yes	Partial Yes	Yes	Yes	Yes	Yes	Yes	Yes	Yes	No	Yes	Yes	Yes	Yes	Yes	Yes	15/16 High
Qiao (2023)(Qiao, Chi, Zhang, & Ma, 2023) [36]	Yes	Partial Yes	Yes	Yes	Yes	Yes	Yes	Yes	Yes	No	Yes	Yes	Yes	Yes	Yes	Yes	15/16 High
Qiao (2024)(Qiao, Gu, Yu, Chi, & Ma, 2024) [35]	Yes	Partial Yes	Yes	Yes	Yes	Yes	Yes	Yes	Yes	No	Yes	Yes	Yes	Yes	Yes	Yes	15/16 High
Shim (2026)(Shim, Lau, Huynh, Lu, & Tan, 2026) [37]	Yes	Yes	Yes	Yes	Yes	Yes	Yes	Yes	Yes	Yes	Yes	Yes	Yes	Yes	Yes	Yes	16/16 High
Su (2025)(Su et al., 2025) [38]	Yes	Partial yes	Yes	Yes	Yes	Yes	Yes	Yes	Yes	Yes	Yes	Yes	Yes	Yes	Yes	Yes	16/16 High
Terao (2024)(Terao & Kodama, 2024) [40]	Yes	Partial yes	Yes	Yes	Yes	Yes	Yes	Yes	Yes	Yes	Yes	Yes	Yes	Yes	Yes	Yes	16/16 High
Wang (2025)(H. Wang, Pan, Zhang, & Tan, 2025) [31]	Yes	Partial yes	No	Yes	Yes	Yes	Partial yes	Partial yes	Yes	No	Yes	Yes	Yes	Yes	Yes	Yes	14/16 Moderate

Wei (2025)(Wei & Li, 2025) [39]	Yes	Yes	Yes	Yes	Yes	Yes	Yes	Yes	Yes	No	Yes	Yes	Yes	Yes	Yes	Yes	15/16 High
Zeng (2024)(Zeng et al., 2024) [32]	Yes	Yes	No	Yes	Yes	Yes	Partial Yes	Yes	Yes	No	Yes	Yes	Yes	Yes	Yes	Yes	14/16 Moderate

Supplementary Table S2. AMSTAR 2 rating for included studies

Questions (Q) 2,4,7,9,11,13,15, shown in bold, are considered critical domains in the AMSTAR-2 scale

Author (Year)	Q1	**Q2**	Q3	**Q4**	Q5	Q6	**Q7**	Q8	**Q9**	Q10	**Q11**	Q12	**Q13**	Q14	**Q15**	Q16	Rating
Abdelazim (2024)	Yes	No	Yes	Partial Yes	Yes	Yes	Partial Yes	Yes	Yes	Yes	Yes	No	Yes	Yes	Yes	Yes	13/16 Low
Alkhalifa (2025)	Yes	No	No	Partial Yes	No	No	No	Partial yes	No	No	N/A	N/A	No	N/A	N/A	Yes	3/12 Critically low
Arroyo-Pacheco (2025)	Yes	No	No	Partial Yes	Yes	Yes	No	Yes	Yes	No	Yes	No	No	Yes	Yes	Yes	10/16 Critically Low
Ashmawy (2025)	Yes	No	No	No	Yes	Yes	No	Yes	Yes	No	Yes	Yes	Yes	Yes	Yes	Yes	11/16 Critically Low
Avgerinos (2024)	Yes	Yes	Yes	Yes	Yes	Yes	No	Yes	Yes	No	Yes	No	Yes	Yes	Yes	Yes	13/16 Low
Cao (2025)	Yes	No	No	Yes	Yes	Yes	No	Yes	Yes	No	Yes	No	No	Yes	Yes	Yes	10/16

																	Critically Low
Chhabra (2024)	Yes	Partial Yes	Yes	Yes	Yes	Yes	Yes	Yes	Yes	No	N/A	N/A	Yes	No	Partial Yes	Yes	12/16 Low
Chowdhury (2023)	Yes	No	Yes	Yes	No	No	Yes	Yes	No	No	N/A	N/A	No	No	Partial Yes	Yes	7/16 Critically Low
Chundu (2025)	Yes	No	Yes	Partial Yes	Yes	Yes	No	Yes	Yes	No	N/A	N/A	Partial Yes	N/A	N/A	Yes	7 / 12 Critically Low
Da Silva (2026)	Yes	Yes	No	Partial Yes	Yes	Yes	No	Yes	Yes	No	Yes	No	Yes	Yes	Yes	Yes	12 /16 Low
Da Silva (2025)	Yes	Yes	No	Partial Yes	No	No	No	Yes	Yes	No	Yes	No	Yes	Yes	Yes	Yes	10 /16 Low
Ebell (2024)	Yes	Yes	Yes	Yes	Yes	Yes	No	Yes	Yes	No	Yes	Yes	Yes	Yes	Yes	Yes	14/16 Low
Elamin (2025)	Yes	No	Yes	Yes	Yes	Yes	Yes	Yes	No	No	N/A	N/A	No	No	No	Yes	8/16 Critically Low
Esquer (2023)	Yes	No	No	Partial Yes	No	No	No	Yes	No	No	N/A	N/A	No	N/A	N/A	Yes	4 /12 Critically Low

Galende (2026)	Yes	No	Yes	Partial Yes	No	No	No	Yes	Yes	No	N/A	N/A	Yes	N/A	N/A	Yes	7 /12 Critically Low
Hossain (2024)	Yes	Yes	No	Partial Yes	Yes	Yes	No	Yes	Yes	No	N/A	N/A	Yes	N/A	N/A	Yes	9 /12 Low
Hu (2025)	Yes	Yes	No	Yes	Yes	Yes	No	Yes	Yes	No	Yes	Yes	Yes	Yes	Yes	Yes	13/16 Low
Huang (2026)	Yes	No	No	Partial Yes	Yes	Yes	No	Yes	Yes	No	Yes	Yes	Yes	Yes	Yes	Yes	12/16 Critically Low
Jeremic (2025)	Yes	No	No	Yes	No	No	Yes	Yes	Yes	No	Yes	Yes	Yes	Yes	Yes	Yes	11/16 Low
Lacorte (2022)	Yes	Yes	No	Yes	Yes	Yes	No	Yes	Yes	No	Yes	No	Yes	Yes	No	Yes	11/16 Low
Liu (2025)	Yes	Yes	No	Partial Yes	Yes	Yes	No	Yes	Yes	No	Yes	No	Yes	Yes	Yes	Yes	12/16 Low
Mostafa (2025)	yes	Partial Yes	Yes	Yes	Yes	Yes	Yes	Yes	No	No	N/A	N/A	No	No	Partial Yes	Yes	10/16 Critically Low
Rashad (2022)	Yes	Partial Yes	Yes	Yes	Yes	Yes	Yes	Yes	Yes	No	N/A	N/A	Yes	No	Yes	Yes	12/16 Low

Tonegawa-Kuji (2025)	Yes	Yes	Yes	Yes	Yes	Yes	No	Yes	Yes	No	Yes	Partial Yes	Yes	Yes	Yes	Yes	14/16 Low
Villain (2022)	Yes	Partial Yes	No	Yes	No	No	Yes	Yes	No	No	Yes	Yes	No	Yes	Yes	Yes	10/16 Critically Low
Wang (2022)	Yes	No	No	No	No	No	No	Yes	No	No	Yes	No	No	Yes	No	Yes	5/16 Critically low
Wu (2023)	Yes	No	No	Partial yes	No	Yes	Partial yes	Yes	Yes	No	Yes	No	Yes	Yes	Yes	Yes	11/16 Low
Zhang (2026)	Yes	No	Yes	No	No	No	No	Partial Yes	No	No	N/A	N/A	No	Yes	N/A	Yes	8/16 Critically low

Questions (Q) 2,4,7,9,11,13,15, shown in bold, are considered critical domains in the AMSTAR2 scale

Supplementary table S3. AMSTAR 2 rating for excluded studies

1 Abdelazim K, Allam AA, Afifi B, Abdulazeem H, Elbehiry AI: The efficacy and safety of lecanemab 10 mg/kg biweekly compared to a placebo in patients with Alzheimer's disease: a systematic review and meta-analysis of randomized controlled trials. Neurological sciences : official journal of the Italian Neurological Society and of the Italian Society of Clinical Neurophysiology 2024, 45(8):3583-3597.

2. Alkhalifa AE, Al Mokhlf A, Ali H, Al-Ghraiybah NF, Syropoulou V: Anti-Amyloid Monoclonal Antibodies for Alzheimer's Disease: Evidence, ARIA Risk, and Precision Patient Selection. J Pers Med 2025, 15(9).

3. Arroyo-Pacheco N, Sarmiento-Blanco S, Vergara-Cadavid G, Castro-Leones M, Contreras-Puentes N: Monoclonal therapy with lecanemab in the treatment of mild Alzheimer's disease: A systematic review and meta-analysis. Ageing research reviews 2025, 104:102620.
4. Ashmawy RE, Okesanya OJ, Ukoaka BM, Daniel FM, Ezedigwe SG, Agboola AO, Ahmed MM, Ogaya JB, Amisu BO, Adigun OA et al: Exploring the efficacy and safety of lecanemab in the management of early Alzheimer's disease: A systematic review of clinical evidence. Journal of Alzheimer's disease : JAD 2025, 105(3):714-728.
5. Avgerinos KI, Manolopoulos A, Ferrucci L, Kapogiannis D: Critical assessment of anti-amyloid-β monoclonal antibodies effects in Alzheimer's disease: a systematic review and meta-analysis highlighting target engagement and clinical meaningfulness. Scientific reports 2024, 14(1):25741.
6. Cao W, Zhu B, Liu Z, Jia X, Zhao H, Gu N, Chang H, Xi J, Li R, Guo K et al: Comparison of the efficacy of updated drugs for the treatment on the improvement of cognitive function in patients with Alzheimer 's disease: A systematic review and network meta- analysis. Neuroscience 2025, 565:29-39.
7. Chhabra A, Solanki S, Saravanabawan P, Venkiteswaran A, Nimmathota N, Modi NM: A systematic review of the efficacy and safety of anti-amyloid beta monoclonal antibodies in treatment of Alzheimer's disease. Expert Opin Biol Ther 2024, 24(11):1261-1269.
8. Chundu UC, Thiriveedhi SR, Bhatti C, Mupparaju JS, Otinashvili N, Gelishvili E: A Systematic Review of the Efficacy and Safety of Anti-amyloid Monoclonal Antibodies in Alzheimer's Disease. Cureus 2025, 17(6):e85377.
9. da Silva AMP, de Deus O, Januário Campos Cardoso L, Virgilio Ribeiro F, Froelich Meldola P, Rodrigues Menezes I, Lee Han M, Quiroga DG, James Almeida K, Haddad Santos D et al: Efficacy, safety, and ARIA risk of anti-β-amyloid antibodies in early Alzheimer's disease: a systematic review, meta-analysis, and meta-regression. Expert Opin Biol Ther 2026, 26(2):177-188.
10. Pereira da Silva AM, Falcão L, Virgilio F, Rodrigues Menezes I, Leite M, Farias E, Nascimento M, Lee Han M, Mota Telles JP, de Souza Franco E et al: Efficacy and APOE ε4-stratified risk of donanemab in Alzheimer's disease: A systematic review and meta-analysis of randomized clinical trials. Journal of Alzheimer's disease : JAD 2025, 107(2):477-493.
11. Ebell MH, Barry HC, Baduni K, Grasso G: Clinically Important Benefits and Harms of Monoclonal Antibodies Targeting Amyloid for the Treatment of Alzheimer Disease: A Systematic Review and Meta-Analysis. Ann Fam Med 2024, 22(1):50-62.
12. Elamin SA, Al Shibli AN, Shaito A, Al-Maadhadi M, Zolezzi M, Pedersen S: Anti-amyloid monoclonal antibody therapies in Alzheimer's disease - a scoping review. Neuroscience 2025, 589:50-61.
13. Esquer A, Blanc F, Collongues N: Immunotherapies Targeting Amyloid and Tau Protein in Alzheimer's Disease: Should We Move Away from Diseases and Focus on Biological Targets? A Systematic Review and Expert Opinion. Neurol Ther 2023, 12(6):1883-1907.
14. Villarejo Galende A, González-Sánchez M, Hilario A, Llamas-Velasco S, Morenas-Rodríguez E, Muñoz-García MI, Ramos-González A, Pérez-Martínez DA, Blanco-Palmero VA: Severe symptomatic amyloid-related imaging abnormalities in Alzheimer's disease: Two case reports and systematic review of reported cases. Journal of Alzheimer's disease : JAD 2026, 109(3):1099-1116.
15. Hossain MF, Husna AU, Kharel M: Use of lecanemab for the treatment of Alzheimer's disease: A systematic review. Brain and behavior 2024, 14(6):e3592.
16. Hu G, Zhang M: Efficacy and Safety of Donanemab in the Treatment of Alzheimer's Disease: A Systematic Review and Meta-Analysis. Current Alzheimer research 2026.

17. Huang S, Guo Y: Network meta-analysis of the efficacy of nine drugs for cognitive function in patients with Alzheimer's disease. J Alzheimers Dis Rep 2026, 10:25424823261422205.
18. Jeremic D, Navarro-Lopez JD, Jimenez-Diaz L: Clinical Benefits and Risks of Antiamyloid Antibodies in Sporadic Alzheimer Disease: Systematic Review and Network Meta-Analysis With a Web Application. J Med Internet Res 2025, 27:e68454.
19. Lacorte E, Ancidoni A, Zaccaria V, Remoli G, Tariciotti L, Bellomo G, Sciancalepore F, Corbo M, Lombardo FL, Bacigalupo I et al: Safety and Efficacy of Monoclonal Antibodies for Alzheimer's Disease: A Systematic Review and Meta-Analysis of Published and Unpublished Clinical Trials. Journal of Alzheimer's disease : JAD 2022, 87(1):101-129.
20. Liu S, Zhao M, Liu Y, Yang X, Yan H, Xu H, Wu Y, Xu Y: Comparative efficacy and safety of symptomatic therapy and disease-modifying therapy for Alzheimer's disease: a systematic review and network meta-analysis. Frontiers in neuroscience 2025, 19:1656906.
21. Mostafa A, Tiu S, Khan F, Baig NA: The Efficacy of Anti-amyloid Monoclonal Antibodies in Early Alzheimer's Dementia: A Systematic Review. Ann Indian Acad Neurol 2025, 28(3):333-343.
22. Rashad A, Rasool A, Shaheryar M, Sarfraz A, Sarfraz Z, Robles-Velasco K, Cherrez-Ojeda I: Donanemab for Alzheimer's Disease: A Systematic Review of Clinical Trials. Healthcare (Basel) 2022, 11(1).
23. Tonegawa-Kuji R, Hou Y, Hu B, Lorincz-Comi N, Pieper AA, Tousi B, Leverenz JB, Cheng F: Efficacy and safety of passive immunotherapies targeting amyloid beta in Alzheimer's disease: A systematic review and meta-analysis. PLoS Med 2025, 22(3):e1004568.
24. Villain N, Planche V, Levy R: High-clearance anti-amyloid immunotherapies in Alzheimer's disease. Part 1: Meta-analysis and review of efficacy and safety data, and medico-economical aspects. Revue neurologique 2022, 178(10):1011-1030.
25. Wang D, Kowalewski EK, Koch G: Application of Meta-analysis to Evaluate Relationships Among ARIA-E Rate, Amyloid Reduction Rate, and Clinical Cognitive Response in Amyloid Therapeutic Clinical Trials for Early Alzheimer's Disease. Ther Innov Regul Sci 2022, 56(3):501-516.
26. Wu W, Ji Y, Wang Z, Wu X, Li J, Gu F, Chen Z, Wang Z: The FDA-approved anti-amyloid-β monoclonal antibodies for the treatment of Alzheimer's disease: a systematic review and meta-analysis of randomized controlled trials. Eur J Med Res 2023, 28(1):544.
27. Zhang B, Yu X, Zhang X: The evolving landscape of amyloid-targeting therapies in Alzheimer's disease: progress and challenges. Neurological sciences : official journal of the Italian Neurological Society and of the Italian Society of Clinical Neurophysiology 2026, 47(3).
